# Crosstalk of PIF4 and DELLA modulates CBF transcript and hormone homeostasis in cold response in tomato

**DOI:** 10.1111/pbi.13272

**Published:** 2019-10-27

**Authors:** Feng Wang, Xiaoxiao Chen, Sangjie Dong, Xiaochun Jiang, Lingyu Wang, Jingquan Yu, Yanhong Zhou

**Affiliations:** ^1^ Department of Horticulture Zhejiang University Hangzhou China; ^2^ Key Laboratory of Plant Growth Development and Quality Improvement Agricultural Ministry of China Hangzhou China; ^3^ Zhejiang Provincial Key Laboratory of Horticultural Plant Integrative Biology Hangzhou China; ^4^ Present address: College of Horticulture Shenyang Agricultural University Shenyang China

**Keywords:** PIF4, cold stress, light signalling, hormone, GAI4, *Solanum lycopersicum* (tomato)

## Abstract

The ability to interpret daily and seasonal fluctuations, latitudinal and vegetation canopy variations in light and temperature signals is essential for plant survival. However, the precise molecular mechanisms transducing the signals from light and temperature perception to maintain plant growth and adaptation remain elusive. We show that far‐red light induces PHYTOCHROME‐INTERACTING TRANSCRIPTION 4 (SlPIF4) accumulation under low‐temperature conditions via phytochrome A in *Solanum lycopersicum* (tomato). Reverse genetic approaches revealed that knocking out *SlPIF4* increases cold susceptibility, while overexpressing *SlPIF4* enhances cold tolerance in tomato plants. SlPIF4 not only directly binds to the promoters of the *C‐REPEAT BINDING FACTOR* (*SlCBF*) genes and activates their expression but also regulates plant hormone biosynthesis and signals, including abscisic acid, jasmonate and gibberellin (GA), in response to low temperature. Moreover, SlPIF4 directly activates the *SlDELLA* gene (*GA‐INSENSITIVE 4*,* SlGAI4*) under cold stress, and *SlGAI4* positively regulates cold tolerance. Additionally, *SlGAI4* represses accumulation of the SlPIF4 protein, thus forming multiple coherent feed‐forward loops. Our results reveal that plants integrate light and temperature signals to better adapt to cold stress through shared hormone pathways and transcriptional regulators, which may provide a comprehensive understanding of plant growth and survival in a changing environment.

## Introduction

As sessile organisms, plants must integrate multiple environmental and endogenous signals to adjust their growth patterns and developmental transitions to withstand adverse environments and minimize damage. Low temperature is a major environmental stress that severely impairs plant growth and productivity and affects the geographical distribution of plants. To survive under cold stress, plants trigger a suite of sophisticated physiological and biochemical processes. Previous studies have revealed that the ICE‐CBF/DREB1 regulatory pathway plays a critical role in cold stress response (Chinnusamy *et al.*, 2007; Thomashow, 1999). The cold stress‐induced CBF transcription factors directly activate the expression of downstream *COLD‐REGULATED* (*COR*) genes and enhance plant cold tolerance. Knocking out all three *CBF* genes leads to an extreme sensitivity to cold stress (Jia *et al.*, 2016; Zhao *et al.*, 2016), while overexpressing *CBFs* leads to constitutively enhanced cold tolerance (Gilmour *et al.*, 2000; Jaglo‐Ottosen *et al.*, 1998).

Light and temperature are not completely independent under natural plant growth conditions; they provide vital immediate and predictive cues for plants to ensure optimal growth and development (Franklin, 2009; Legris *et al.*, 2017). It has been reported that light is essential for the development of cold acclimation in plants (Kim *et al.*, 2002). Furthermore, the circadian clock, photoperiod and light quality also regulate plant cold tolerance (Dong *et al.*, 2011; Franklin and Whitelam, 2007; Lee and Thomashow, 2012; Li *et al.*, 2016b; Wang *et al.*, 2016, 2018, 2019). *CIRCADIAN CLOCK‐ASSOCIATED 1* (*CCA1*)‐mediated and *LATE‐ELONGATED HYPOCOTYL* (*LHY*)‐mediated outputs from the circadian clock positively regulate plant cold tolerance through the CBF pathway in *Arabidopsis* (Dong *et al.*, 2011). Meanwhile, the CBF pathway is actively repressed by PHYTOCHROME‐INTERACTING FACTOR 4 (PIF4) and PIF7 during the warm long‐day season in *Arabidopsis* (Lee and Thomashow, 2012). Blue light and low temperature‐induced COR27 and COR28 negatively regulate freezing tolerance in *Arabidopsis* (Li *et al.*, 2016b), whereas a low red/far‐red light ratio (L‐R/FR) induces cold tolerance in both *Arabidopsis* and *Solanum lycopersicum* (Franklin and Whitelam, 2007; Wang *et al.*, 2016, 2018). Intriguingly, recent work has demonstrated that *Arabidopsis* phytochrome B (phyB) acts as a thermosensor (Jung *et al.*, 2016; Legris *et al.*, 2016), and it negatively regulates cold tolerance in both *Arabidopsis* and tomato (Franklin and Whitelam, 2007; Wang *et al.*, 2016, 2018). Although we found that phytochrome A (phyA) and phyB function antagonistically to regulate cold tolerance in tomato (Wang *et al.*, 2016), whether phyA or other photoreceptors are sensors for low temperature remains to be investigated. LONG HYPOCOTYL 5 (HY5), a bZIP transcription factor, acts downstream of phytochromes and integrates light and cold signalling to optimize plant survival under cold stress (Catalá *et al.*, 2011; Wang *et al.*, 2018, 2019). Therefore, plants have evolved a delicate system that perceives light and temperature signals, allowing them to exquisitely detect and predict changes in the natural environment (Franklin, 2009). PIFs are basic helix–loop–helix transcription factors and have key roles in light‐regulated plant development and plant responses to multiple environmental signals (Leivar and Monte, 2014; Leivar and Quail, 2011; Pham *et al.*, 2018). PIFs, particularly PIF4, have emerged as a central signalling hub controlling the thermosensory activation of flowering (Kumar *et al.*, 2012) and thermosensory growth in *Arabidopsis* (Delker *et al.*, 2014; Gangappa and Kumar, 2017). However, the evening‐expressed clock component AtTOC1 interacts with and inactivates AtPIF4 to suppress thermoresponsive growth in the evening, which may serve to increase fitness by matching thermoresponsiveness with the day–night cycles of fluctuating temperature and light conditions (Zhu *et al.*, 2016). AtPIFs also coordinate light and temperature to regulate the transcription of photosynthesis and photoprotection genes (Toledo‐Ortiz *et al.*, 2014). In the natural environment, there is a significant drop in the R/FR ratio during shade and twilight periods in autumn months (Casal and Qüesta, 2018; Ross *et al.*, 1986). L‐R/FR stabilized the AtPIF4 protein during shade avoidance (Lorrain *et al*., 2008), and we previously demonstrated that L‐R/FR induced cold tolerance in tomato shade leaves (Wang *et al.*, 2018). Therefore, whether PIF4 is the central signalling hub that integrates light and temperature to regulate cold tolerance in tomato remains to be explored.

PIFs are emerging as integrators of signals from different hormone pathways during growth and development (Leivar and Monte, 2014). Recent studies have demonstrated that AtPIF4/PIF5 induces ethylene and abscisic acid (ABA) signalling in leaf senescence (Sakuraba *et al.*, 2014). Hormones in the gibberellin (GA) and brassinosteroid (BR) classes are also involved in AtPIF4‐mediated light and temperature signalling (Franklin *et al.*, 2014). DELLA proteins are the key repressors of almost all GA responses (Ueguchi‐Tanaka *et al.*, 2007). DELLAs interact with PIFs and have a dual role in modulating PIFs by both sequestration and degradation (Li *et al.*, 2016a). It has been demonstrated that plant hormones, such as ABA, jasmonate (JA), BR and GA, are involved in plant cold tolerance (Achard *et al.*, 2008; Li *et al.*, 2017; Wang *et al.*, 2016; Zhou *et al.*, 2017). Therefore, plants enhance the capacity of perception and prediction of seasonal changes by the multiple integration of light and temperature signals with hormone‐signalling pathways and transcriptional regulators (Franklin, 2009).

In this study, we show that far‐red light (FR) induces SlPIF4 accumulation dependent on phyA under low‐temperature conditions in *Solanum lycopersicum*. SlPIF4 positively regulates plant cold tolerance in tomato by directly binding to the promoters of the *SlCBF* genes and activating their expression, while promoting ABA and JA signalling under cold stress. SlPIF4 also directly associates with the promoter sequence of *SlGAI4*, which encodes a DELLA protein in tomato, and activates its expression under cold stress. Interestingly, when large amounts of SlGAI4 protein accumulated during cold stress, it repressed SlPIF4 accumulation in a negative feedback manner. Thus, our results suggest that SlPIF4 is a pivotal component of light and temperature cues and integrates environmental stimuli with plant hormones to coordinate tomato plant growth with impending cold temperatures.

## Results

### Far‐red light and low temperature induce SlPIF4 accumulation via a phytochrome‐dependent pathway in tomato

**Figure 1:**
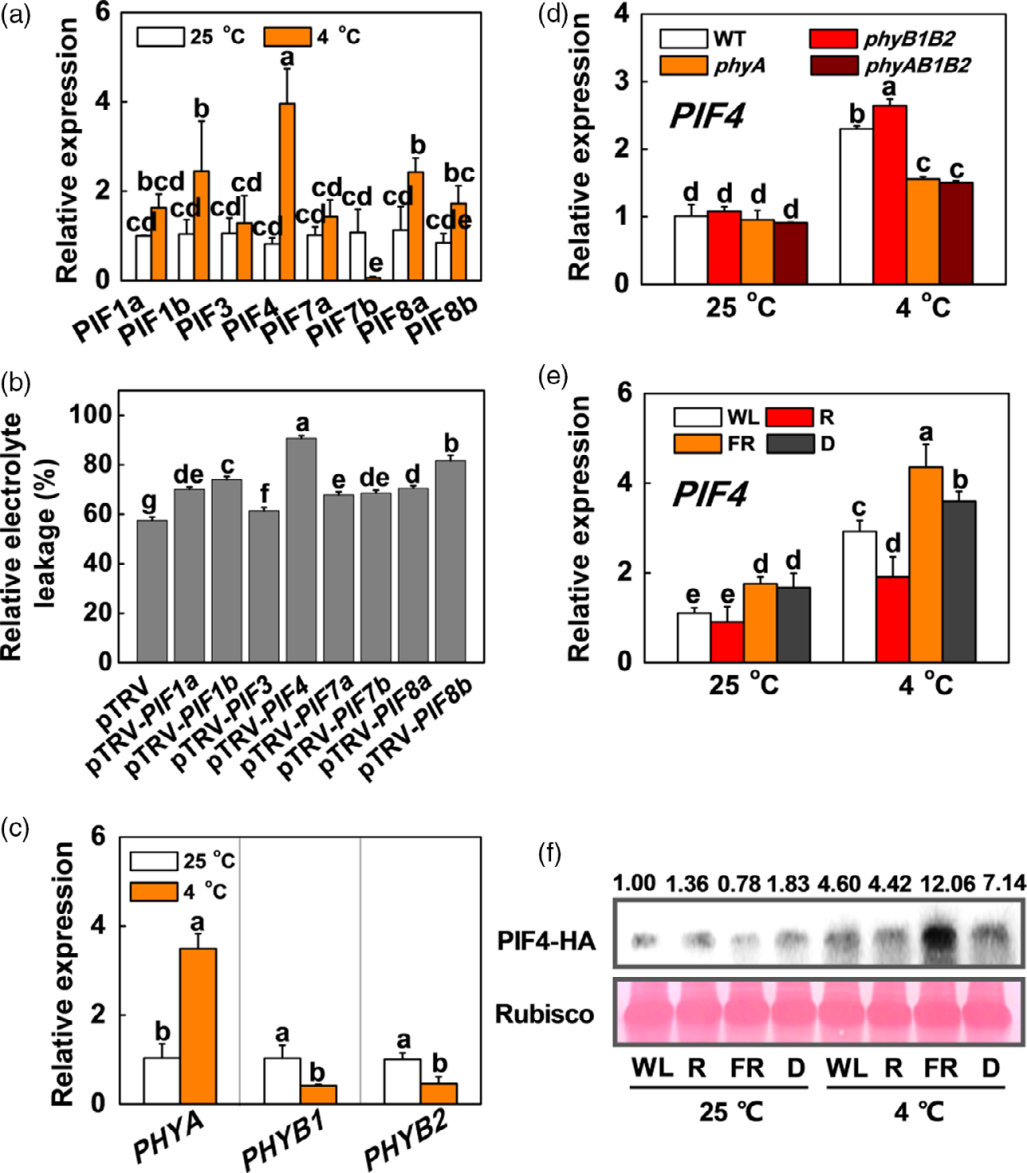
SlPIF4 is regulated by both light and low temperature. (a) and (b) Expression of *SlPIFs* in tomato wild‐type (WT) plants (a) and REL in *SlPIF*‐silenced plants (b) grown at white light (120 µmol m^−2^ s^−1^) after exposure to 4 °C for 6 h and 7 days, respectively. (c) Expression of *PHYA*, *PHYB1* and *PHYB2* in tomato plants after exposure to 25 °C or 4 °C for 6 h. (d) Expression of *SlPIF4* in tomato WT plants and phytochrome mutants (*phyA*, *phyB1B2* and *phyAB1B2*) after exposure to 25 °C or 4 °C for 6 h under white light conditions (120 µmol m^−2^ s^−1^). (e) Expression of *SlPIF4* in WT plants after exposure to 25 °C or 4 °C for 6 h, which grown under dark (D), white light (WL), red light (R) or FR light conditions. The light intensity is 120 µmol m^−2^ s^−1^. (f) Accumulation of SlPIF4 protein in tomato *SlPIF4*‐overexpressing (*SlPIF4‐*OE) plants after exposure to 25 °C or 4 °C for 12 h, which grown under D, WL, R or FR conditions. The light intensity is 120 µmol m^−2^ s^−1^. Data are presented as the means of three biological replicates (±SD). Different letters indicate significant differences (*P* < 0.05) according to Tukey’s test.

To investigate the possible involvement of SlPIFs in the plant response to cold stress, we identified the eight tomato *SlPIF* genes through phylogenetic analysis (Figure S1) and investigated the expression of these genes in tomato plants exposed to cold stress. We found that the expression of *SlPIF4* was the highest among the eight *SlPIF* genes after the plants were exposed to 4 °C (~2‐fold than other genes; Figure 1a). To clarify which *SlPIF* gene is the major gene in response to cold stress, we silenced *SlPIF* family genes by tobacco rattle virus‐induced gene silencing (VIGS). After cold stress, the relative electrolyte leakage (REL) in *SlPIF4*‐silenced plants (pTRV‐*PIF4*) was higher than those of the other *SlPIF* gene‐silenced plants (Figure 1b), which further demonstrated that *SlPIF4* was the predominant gene among the SlPIF family genes in response to cold stress. Since low temperature induced *SlPHYA* gene expression, but inhibited transcripts of *SlPHYB1* and *SlPHYB2* compared with those in plants grown at 25 °C (Figure 1c), we then wanted to know whether the regulation of *SlPIF4* by low temperature was dependent on phytochrome. The results showed that *SlPIF4* expression was higher in tomato *phyB* mutants than in wild‐type (WT) plants, while its expression was lower in tomato *phyA* and *phyAB1B2* mutants than in WT plants under cold stress (Figure 1d), which indicated that low temperature regulated *SlPIF4* via the phytochrome pathway. Since FR enhanced cold tolerance via phyA, and R inhibited cold tolerance via phyB in tomato plants (Wang *et al.*, 2016), we then asked whether SlPIF4 was regulated by light quality during cold stress. We examined the gene expression of *SlPIF4* and its protein accumulation in tomato plants under different light conditions, such as white light (WL), red light (R), FR light and dark (D). Compared with plants grown at 25 °C, plants grown at a low temperature had markedly induced *SlPIF4* gene expression and protein accumulation, especially in combination with FR conditions (Figure 1e,f). The gene expression and protein accumulation of SlPIF4 increased and decreased in plants under FR and R conditions, respectively, compared with those in plants under WL conditions at 4 °C (Figure 1e,f). These results suggest that R and FR function antagonistically to regulate SlPIF4 accumulation via a phytochrome‐dependent pathway in tomato plants.

### SlPIF4 is a positive regulator in L‐R/FR‐induced plant cold tolerance and directly activates *CBF* gene expression

**Figure 2:**
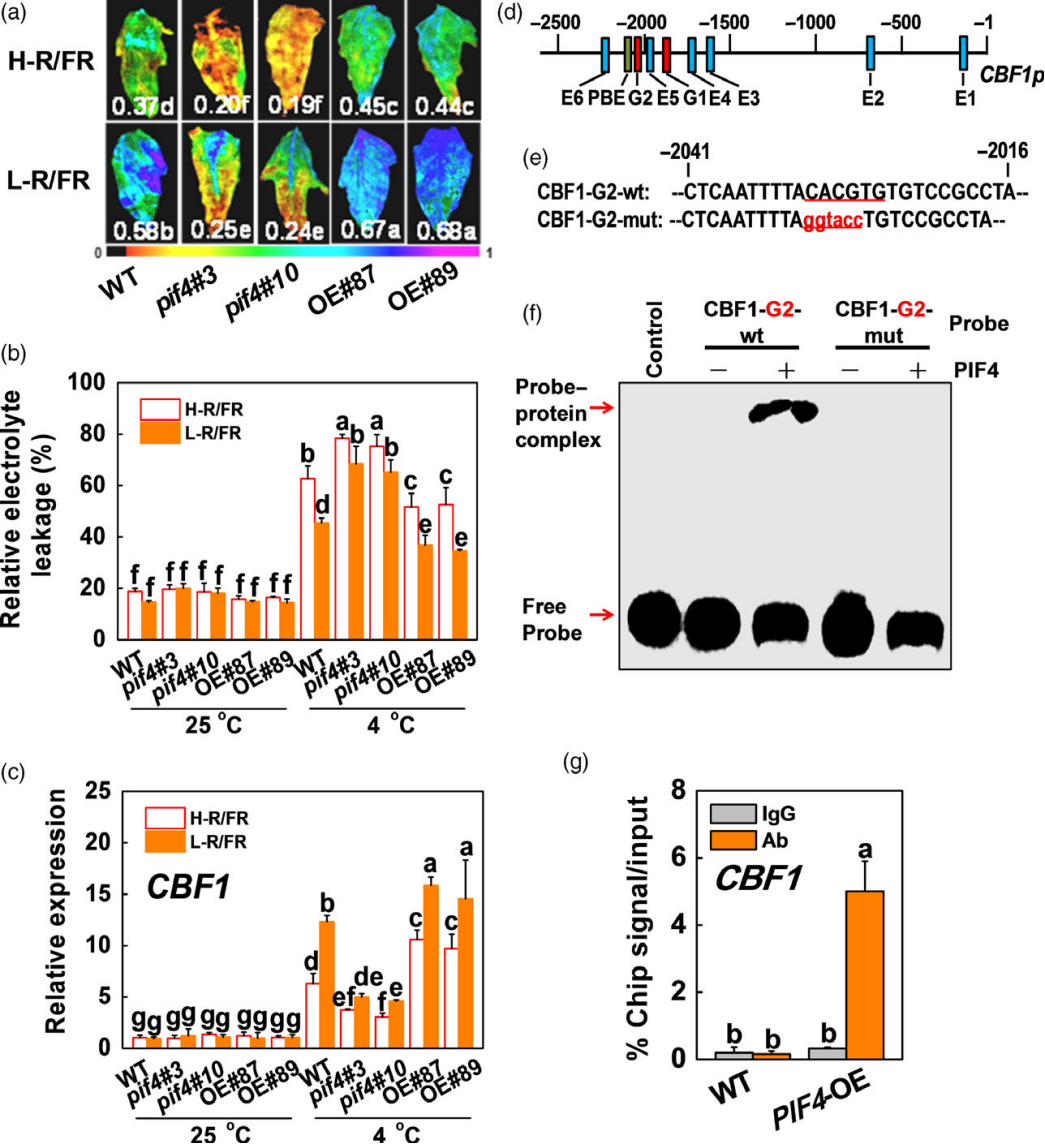
SlPIF4 positively regulates cold tolerance in tomato and directly activates *SlCBF1* expression. (a) Fv/Fm in tomato wild‐type (WT), *pif4* mutant (*pif4*#3, *pif4*#10) and *SlPIF4*‐OE (OE#87, OE#89) plants after exposure to 4 °C under high R/FR (H‐R/FR, 2.5) light or low R/FR (L‐R/FR, 0.5) light for 7 days. The false‐colour code depicted at the bottom of the image ranges from 0 (black) to 1.0 (purple), representing the level of damage in the leaves. (b) and (c) REL (b) and *SlCBF1* gene expression (c) in tomato WT, *pif4* mutant (*pif4*#3, *pif4*#10) and *SlPIF4*‐OE (OE#87, OE#89) plants after exposure to 25 °C or 4 °C under H‐R/FR or L‐R/FR conditions for 7 days and 6 h, respectively. (d) and (e) G‐, E‐ and PBE‐box elements in the promoter of tomato *SlCBF1* gene (d) and oligonucleotide used in the EMSA (e). Numbering is from predicted transcriptional start sites. The G‐box core sequence was mutated in the CBF1‐G2‐mut probe. The wt and mutated G‐box sequences are underlined. The mutated bases were indicated in red. (f) EMSA. The His‐SlPIF4 recombinant protein was incubated with biotin‐labelled wild‐type (CBF1‐G2‐wt) or mutant (CBF1‐G2‐mut) oligos. The protein purified from the empty vector was used as a negative control. (g) ChIP‐qPCR assay. WT and *35S:SlPIF4*‐HA tomato plants were grown at 4 °C under L‐R/FR light for 6 h, and samples were precipitated with an anti‐HA antibody. A control reaction was processed simultaneously using mouse IgG. The ChIP results are presented as percentages of the input DNA. For light‐quality treatments, plants were maintained at R conditions (120 µmol m^−2^ s^−1^) and supplemented with different intensities of FR. Data are presented as the means of three biological replicates (±SD). Different letters indicate significant differences (*P* < 0.05) according to Tukey’s test.

Since there is a significant drop in the R/FR ratio during twilight periods in autumn months, we used white light supplemented with R and/or FR to obtain different R/FR ratios and examined the effects of different R/FR ratios on cold tolerance in tomato. We found that low R/FR light ratios (L‐R/FR) could alleviate cold‐induced leaf wilted, the increased REL and the decreased maximum photochemical efficiency of PSII (Fv/Fm) compared with high R/FR light ratios (H‐R/FR; Figure S2). To determine the role of SlPIF4 in L‐R/FR‐induced cold tolerance, we generated *pif4* mutant and *SlPIF4*‐overexpressing (*SlPIF4*‐OE) transgenic tomato plants. Two independent *pif4* mutants (*pif4*#3 and *pif4*#10) and two independent overexpression lines of tomato (OE#87 and OE#89) were used for further analysis (Figure S3), along with the corresponding untransformed WT. We found that D induced the tomato hypocotyl length compared with WL, but the hypocotyl length of *pif4* mutant and *SlPIF4*‐OE was the same in tomato under WL and D conditions (Figure S4). However, we found that the *pif4* mutant exhibited increased sensitivity to cold stress, while the *SlPIF4*‐OE plants exhibited decreased sensitivity to cold stress, as indicated by the changes in Fv/Fm and REL (Figures 2a,b and S5a). The Fv/Fm values and REL were lower and higher in the *pif4* mutant, respectively, than those in WT, while the Fv/Fm values and REL in the *SlPIF4*‐OE plants were higher and lower, respectively, than those of the WT plants after cold stress (Figure 2a,b). Meanwhile, the leaves were more wilted in the *pif4* mutant, but less wilted in the *SlPIF4*‐OE plants, respectively, than those in WT after cold stress (Figure S5a). These results indicate that SlPIF4 positively regulates cold tolerance in tomato plants. In addition, we found L‐R/FR decreased REL and increased the Fv/Fm values in both the WT and *SlPIF4*‐OE plants under cold stress, but these positive effects on cold tolerance were almost abolished in the tomato *pif4* mutant. Meanwhile, L‐R/FR induced the transcription of *SlCBF1* and *SlCOR413‐like* in the WT and *SlPIF4*‐OE plants was mostly abolished in the tomato *pif4* mutant plants (Figures 2c and S5b). These results indicate that L‐R/FR‐induced cold tolerance is partially dependent on SlPIF4.

Next, we investigated whether SlPIF4 directly regulated the transcription of *SlCBF1*. A previous study showed that the PIF proteins recognize the G‐box, E‐box and PBE‐box motifs (Kim *et al.*, 2003), and promoter analysis revealed the presence of G‐box, E‐box and PBE‐box motifs in the *SlCBF1* gene promoter (Figure 2d). Thus, we performed electrophoretic mobility shift assays (EMSAs) to test whether SlPIF4 could directly bind to these fragments of the *SlCBF1* gene promoter *in vitro*. EMSA showed that the His‐SlPIF4 protein bound directly to the biotin‐labelled G‐box‐containing probe (G2‐box) of the *SlCBF1* promoter (nucleotides −2041 to −2016) and caused a mobility shift (Figure 2e,f). Mutation of the core sequence of the G‐box motif in the *SlCBF1* probes (SlCBF1‐G2‐mut) resulted in the loss of the capacity of SlPIF4 to bind the probes. Then, we performed chromatin immunoprecipitation (ChIP) assays to test whether SlPIF4 was associated with the *CBF* promoters *in vivo*. The qPCR data showed that the promoters of *SlCBF1*, *SlCBF2* and *SlCBF3* were significantly enriched in the *35S:SlPIF4*‐HA samples compared with the WT control, whereas the IgG control was not enriched (Figures 2g and S6). These results indicate that SlPIF4 directly binds to the G‐box motifs in the *SlCBF1* gene promoters. Collectively, these data demonstrate that SlPIF4 positively regulates cold tolerance by directly binding to the promoters of the *SlCBF* genes and activating their transcription in response to cold stress.

### SlPIF4 promotes ABA and JA biosynthesis but inhibits GA biosynthesis in response to cold stress

**Figure 3:**
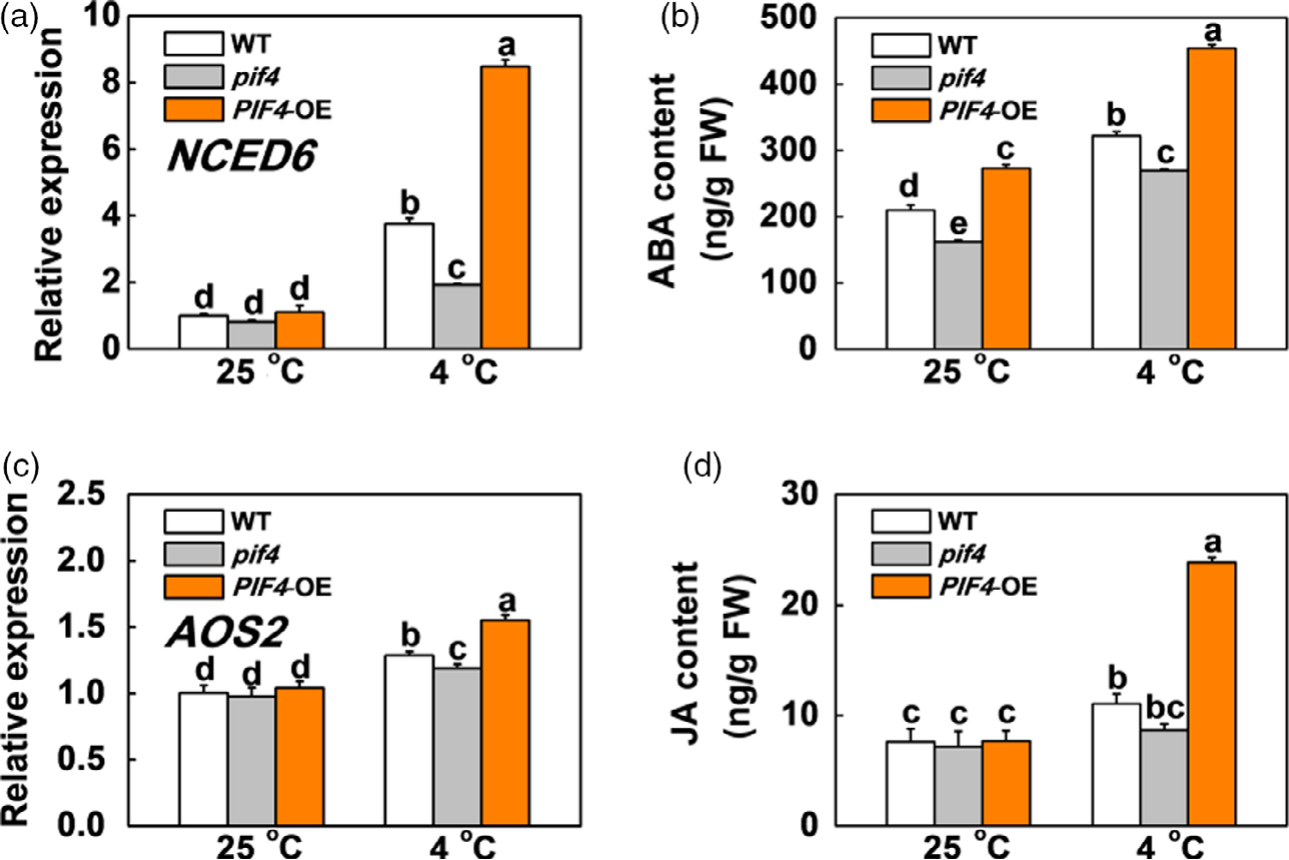
SlPIF4 promotes the gene expression and endogenous levels of ABA and JA biosynthesis in response to cold stress. (a) and (c) Expression of *NCED6* (a) and *AOS2* (c) in tomato WT, *pif4* mutant and *SlPIF4*‐OE plants after exposure to 25 °C or 4 °C under low R/FR (L‐R/FR, 0.5) light conditions for 6 h. (b) and (d) Endogenous levels of ABA (b) and JA (d) biosynthesis in tomato WT, *pif4* mutant and *SlPIF4*‐OE plants after exposure to 25 °C or 4 °C under L‐R/FR light for 12 h. For light‐quality treatments, plants were maintained at R conditions (120 µmol m^−2^ s^−1^) and supplemented with different intensities of FR. Data are presented as the means of three biological replicates (±SD). Different letters indicate significant differences (*P* < 0.05) according to Tukey’s test.

**Figure 4:**
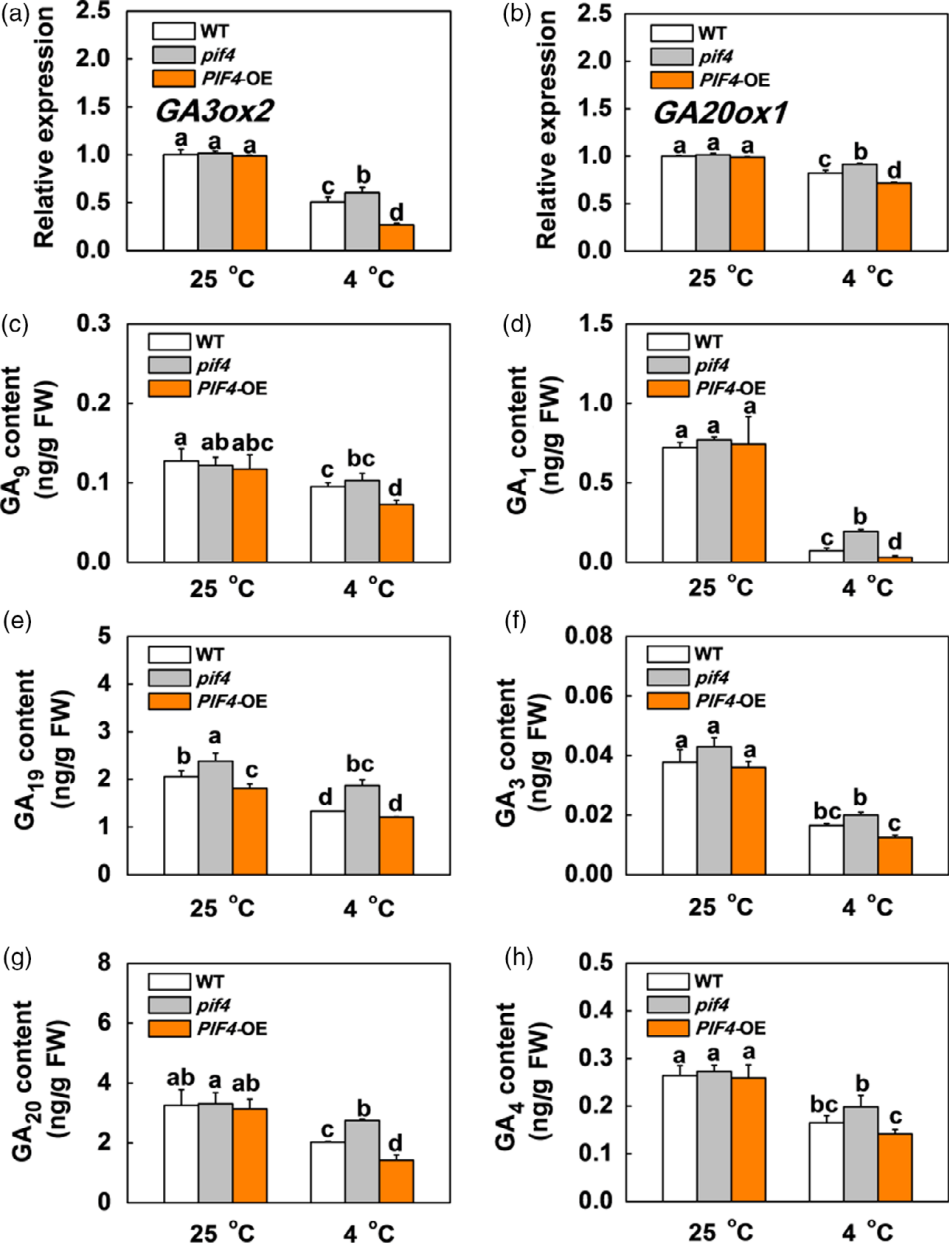
SlPIF4 negatively regulates expression of GA biosynthesis genes and endogenous levels of GA under cold stress. (a) and (b) Expression of *GA3ox2* (a) and *GA20ox1* (b) in tomato WT, *pif4* mutant and *SlPIF4*‐OE plants after exposure to 25 °C or 4 °C under low R/FR (L‐R/FR, 0.5) light conditions for 6 h. (c) to (h) Levels of active GAs (GA_1_, GA_3_ and GA_4_; d, f and h), their precursors (GA_9_, GA_19_ and GA_20_; c, e and g) in tomato WT, *pif4* mutant and *SlPIF4*‐OE plants after exposure to 25 °C or 4 °C under L‐R/FR light for 12 h. For light‐quality treatments, plants were maintained at R conditions (120 µmol m^−2^ s^−1^) and supplemented with different intensities of FR. Data are presented as the means of three biological replicates (±SD). Different letters indicate significant differences (*P* < 0.05) according to Tukey’s test.

Abscisic acid and JA have been shown to enhance plant cold tolerance, while GA inhibits plant cold tolerance (Achard *et al.*, 2008; Wang *et al.*, 2016). We found that the transcription levels of genes involved in ABA biosynthesis (*SlNCED6*) and signalling (*SlAREB*), and JA biosynthesis (*SlAOS2*) and signalling (*SlCOI1*) were not significantly different between the WT, *pif4* mutant and *SlPIF4*‐OE plants at 25 °C (Figures 3a,c and S7). In contrast, these genes were markedly up‐regulated after cold stress, especially in the *SlPIF4*‐OE plants. The transcript levels of these genes were lower in the *pif4* mutant plants than in the WT plants under cold stress. We also noted greater increases in ABA and JA accumulation in the leaves of the WT and *SlPIF4*‐OE plants than the *pif4* mutant plants after cold stress, especially in the *SlPIF4*‐OE plants (Figure 3b,d). In contrast, the transcript levels of GA biosynthesis genes (*SlGA3ox2* and *SlGA20ox1*) and the levels of active GAs (GA_1_, GA_3_ and GA_4_) and their precursors (GA_9_, GA_19_ and GA_20_) significantly decreased after cold stress (Figure 4). Meanwhile, the transcript levels of these GA biosynthesis genes and the accumulation levels of these GAs were higher in the *pif4* mutant plants and lower in the *SlPIF4*‐OE plants than in the WT plants at 4 °C (Figure 4). These results indicate that SlPIF4 enhances cold tolerance in tomato plants partially by inducing ABA and JA, and repressing GA biosynthesis.

### SlPIF4 directly binds the promoter of *SlGAI4* and activates its transcription during cold stress

**Figure 5:**
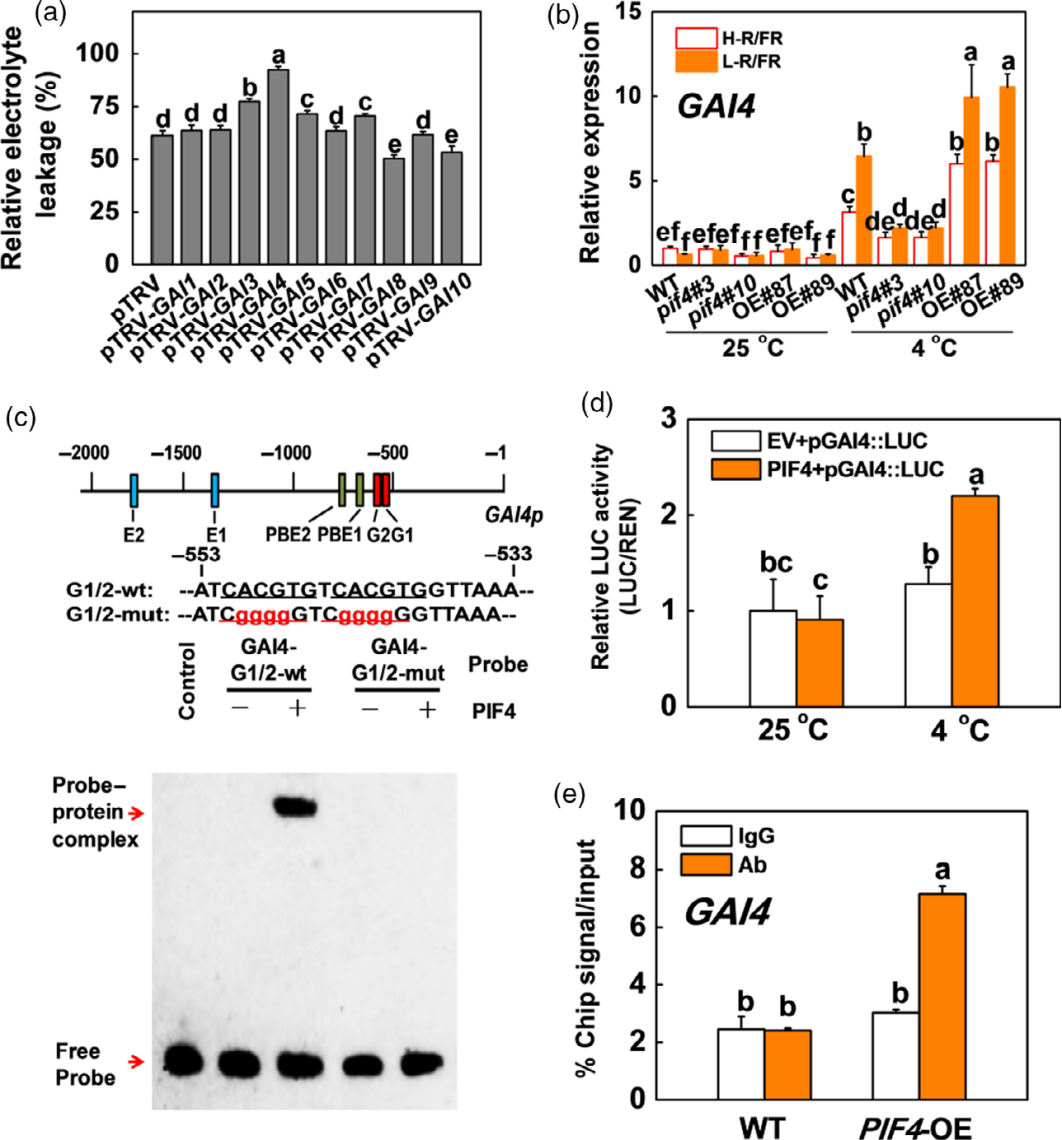
SlPIF4 directly binds to the promoter of *SlGAI4* and activates its expression under cold stress in tomato. (a) The REL in tomato wild‐type (pTRV) and *SlGAI*‐silenced (pTRV‐*GAI1*, pTRV‐*GAI2*, pTRV‐*GAI3*, pTRV‐*GAI4*, pTRV‐*GAI5*, pTRV‐*GAI6*, pTRV‐*GAI7*, pTRV‐*GAI8*, pTRV‐*GAI9*, pTRV‐*GAI10*) plants after exposure to 4 °C for 7 days. (b) Expression of *SlGAI4* in tomato WT, *pif4* mutant (*pif4*#3, *pif4*#10) and *SlPIF4*‐OE (OE#87, OE#89) plants after exposure to 25 °C or 4 °C under high R/FR (H‐R/FR, 2.5) light or low R/FR (L‐R/FR, 0.5) light conditions for 6 h. (c) EMSA. G‐, E‐ and PBE‐box elements in the promoter of tomato *SlGAI4* gene and oligonucleotide used in the EMSA. Numbering is from predicted transcriptional start sites. The G‐box core sequence was mutated in the GAI4‐G1/2‐mut probe. The wt and mutated G‐box sequences are underlined. The mutated bases were indicated in red. The His‐SlPIF4 recombinant protein was incubated with biotin‐labelled wild‐type (GAI4‐G1/2‐wt) or mutant (GAI4‐G1/2‐mut) oligos. The protein purified from the empty vector was used as a negative control. (d) Dual‐LUC assay showing the effects of SlPIF4 on *SlGAI4* promoter activation under cold stress. The *SlGAI4* promoter was fused to the luciferase (LUC) reporter (pGAI4::LUC), and promoter activity was determined by transient expression of it with empty vector (EV) or 35S:*SlPIF4* (PIF4) in tobacco. The tobacco plants were exposed to 25 °C or 4 °C for 24 h after infiltration at 25 °C for 24 h. Relative LUC activity was normalized to the *Renilla* (REN) luciferase. (e) ChIP‐qPCR assay. WT and *35S:SlPIF4*‐HA tomato plants were grown at 4 °C under L‐R/FR light for 6 h, and samples were precipitated with an anti‐HA antibody. A control reaction was processed simultaneously using mouse IgG. The ChIP results are presented as percentages of the input DNA. For light‐quality treatments, plants were maintained at R conditions (120 µmol m^−2^ s^−1^) and supplemented with different intensities of FR. Data are presented as the means of three biological replicates (±SD). Different letters indicate significant differences (*P* < 0.05) according to Tukey’s test.

DELLA proteins are the key repressors of almost all GA responses (Daviere and Achard, 2016) and play critical roles in the plant cold response (Achard *et al.*, 2008; Zhou *et al.*, 2017). There are ten *DELLA* genes (*GA‐INSENSITIVE*, *SlGAIs*) in tomato, which were identified by phylogenetic analysis of tomato DELLA family genes (Figure S8a). VIGS experiments showed that the REL in *SlGAI4*‐silenced plants (pTRV‐*GAI4*) was higher than those of the other *SlGAI*‐silenced plants (Figure 5a), which demonstrated that *SlGAI4* was the predominant gene among the SlGAI family genes responsible for plant cold tolerance. To determine whether SlPIF4 participated in the regulation of *SlGAI4*, we analysed the expression levels of the *SlGAI4* gene in the WT, *pif4* mutant and *SlPIF4*‐OE plants at 25 and 4 °C (Figure 5b). The transcription of *SlGAI4* was induced by low temperatures, especially under L‐R/FR conditions, with the *pif4* and the *SlPIF4*‐OE plants exhibiting lower and higher transcript levels of *SlGAI4*, respectively, than the WT plants (Figure 5b). Promoter analysis revealed that there were two G‐box‐, two E‐box‐ and two PBE‐box‐containing fragments in the promoter of the *SlGAI4* gene (Figure 5c). EMSA showed that SlPIF4 bound to the biotin‐labelled probes containing G‐boxes (nucleotides −553 to −533), leading to a mobility shift, but the capacity to bind to the *SlGAI4* promoter was lost when the promoter was mutated in the G‐box elements (GAI4‐G1/2‐mut; Figure 5c). Dual‐luciferase assays indicated that SlPIF4 significantly activated the promoter of *SlGAI4* under low‐temperature conditions (Figures 5d and S8b). The results were further verified with ChIP assays, which showed that the *SlGAI4* promoter sequence was significantly enriched in the *35S:SlPIF4*‐HA (*SlPIF4*‐OE) samples pulled down by the anti‐HA antibody compared with the WT control samples. No enrichment of the IgG control was observed (Figure 5e). Therefore, SlPIF4 directly binds to the promoter sequence of *SlGAI4* and activates its gene expression during cold stress.

### *SlGAI4* is a positive regulator in L‐R/FR‐induced plant cold tolerance

**Figure 6:**
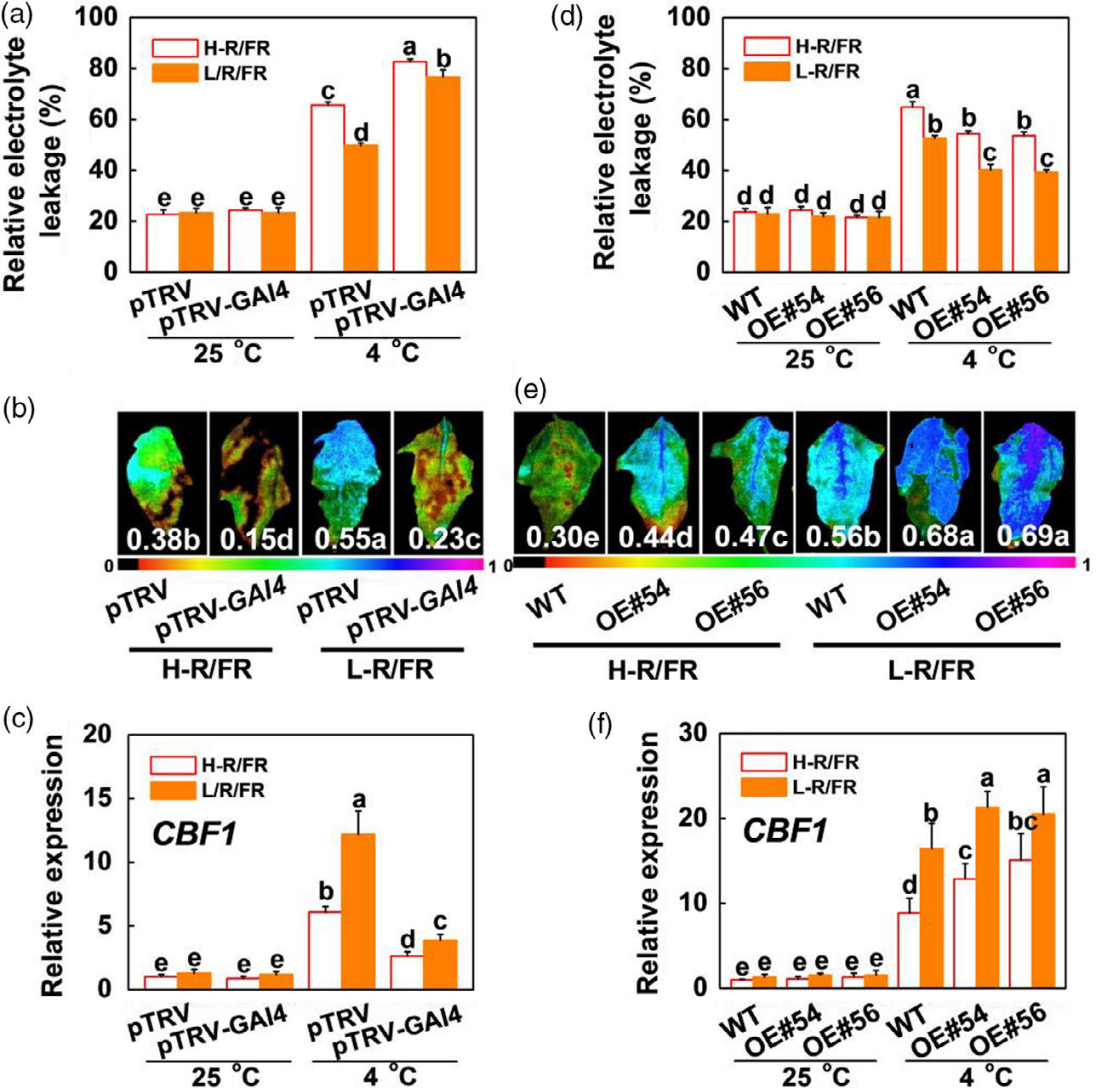
*SlGAI4* is a positive regulator in L‐R/FR‐induced cold tolerance in tomato. (a) The REL in tomato wild‐type (pTRV) and *SlGAI4*‐silenced plants (pTRV‐*GAI4*) after exposure to 25 °C or 4 °C under high R/FR (H‐R/FR, 2.5) light or low R/FR (L‐R/FR, 0.5) light conditions for 7 days. (b) Fv/Fm in tomato pTRV and pTRV‐*GAI4* plants after exposure to 4 °C under H‐R/FR or L‐R/FR conditions for 7 days. The false‐colour code depicted at the bottom of the image ranges from 0 (black) to 1.0 (purple), representing the level of damage in the leaves. (c) Expression of *SlCBF1* in tomato pTRV and pTRV‐*GAI4* plants after exposure to 25 °C or 4 °C under H‐R/FR or L‐R/FR conditions for 6 h. (d) The REL in tomato wild‐type (WT) and *SlGAI4*‐overexpressing plants (OE#54, OE#56) after exposure to 25 °C or 4 °C under H‐R/FR or L‐R/FR conditions for 7 days. (e) Fv/Fm in tomato WT and *SlGAI4*‐overexpressing plants (OE#54, OE#56) plants after exposure to 4 °C under H‐R/FR or L‐R/FR conditions for 7 days. (f) Expression of *SlCBF1* in tomato WT and *SlGAI4*‐overexpressing plants (OE#54, OE#56) plants after exposure to 25 °C or 4 °C under H‐R/FR or L‐R/FR conditions for 6 h. For light‐quality treatments, plants were maintained at R conditions (120 µmol m^−2^ s^−1^) and supplemented with different intensities of FR. Data are presented as the means of three biological replicates (±SD). Different letters indicate significant differences (*P* < 0.05) according to Tukey’s test.

To substantiate the role of *SlGAI4* in cold tolerance, we obtained *SlGAI4*‐silenced tomato plants (pTRV‐*SlGAI4*) and *SlGAI4*‐overexpressing plants (*SlGAI4*‐OE#54, *SlGAI4*‐OE#56) and analysed the expression levels of *SlGAI4* in these plants by qRT‐PCR. The results showed that *SlGAI4* gene transcription was suppressed by 62% and induced by ~20‐fold in *SlGAI4*‐silenced plants and *SlGAI4*‐overexpressing plants, respectively (Figure S9). Then, the *SlGAI4*‐silenced plants (pTRV‐*SlGAI4*), pTRV plants, *SlGAI4*‐overexpressing plants and WT plants were exposed to cold stress under different light conditions (H‐R/FR or L‐R/FR). No differences in REL were observed between the pTRV‐*SlGAI4* and pTRV plants and between the WT and *SlGAI4*‐overexpressing plants grown under optimal growth conditions (Figure 6a,d). However, the pTRV‐*SlGAI4* plants showed increased sensitivity to cold stress at 4 °C, with wilted leaf phenotypes, and L‐R/FR‐induced cold tolerance was also significantly decreased at 4 °C in the pTRV‐*SlGAI4* plants compared with the pTRV plants, as indicated by the increased REL and decreased Fv/Fm values (Figures 6a,b and S10a). In contrast, the REL of the *SlGAI4*‐overexpressing plants decreased significantly, and Fv/Fm values increased consistently compared with those of the WT plants at 4 °C (Figures 6d,e and S10a). These results demonstrate that *SlGAI4* is a positive regulator in L‐R/FR‐induced cold tolerance in tomatoes.

We further examined the expression levels of the cold stress‐responsive genes, such as *SlCBF1* and *SlCOR413‐like*, in the *SlGAI4*‐silenced plants (pTRV‐*SlGAI4*), *SlGAI4*‐overexpressing plants and the WT plants (pTRV and WT) via qRT‐PCR. The expression levels of *SlCBF1* and *SlCOR413‐like* were significantly higher in the WT and *SlGAI4*‐overexpressing plants under cold stress, especially in the *SlGAI4*‐overexpressing plants, whereas those of *SlCBF1* and *SlCOR413‐like* were lower in the pTRV‐*SlGAI4* plants than in the pTRV plants (Figures 6c,f and S10b,c). Furthermore, L‐R/FR‐induced *SlCBF1* and *SlCOR413‐like* gene expression levels in the WT and *SlGAI4*‐overexpressing plants were markedly decreased in the *SlGAI4*‐silenced plants. These results indicate that *SlGAI4* positively regulates the CBF‐pathway genes under cold stress.

### *SlGAI4* acts downstream of SlPIF4 to positively regulate ABA and JA biosynthesis and signalling under cold stress

**Figure 7:**
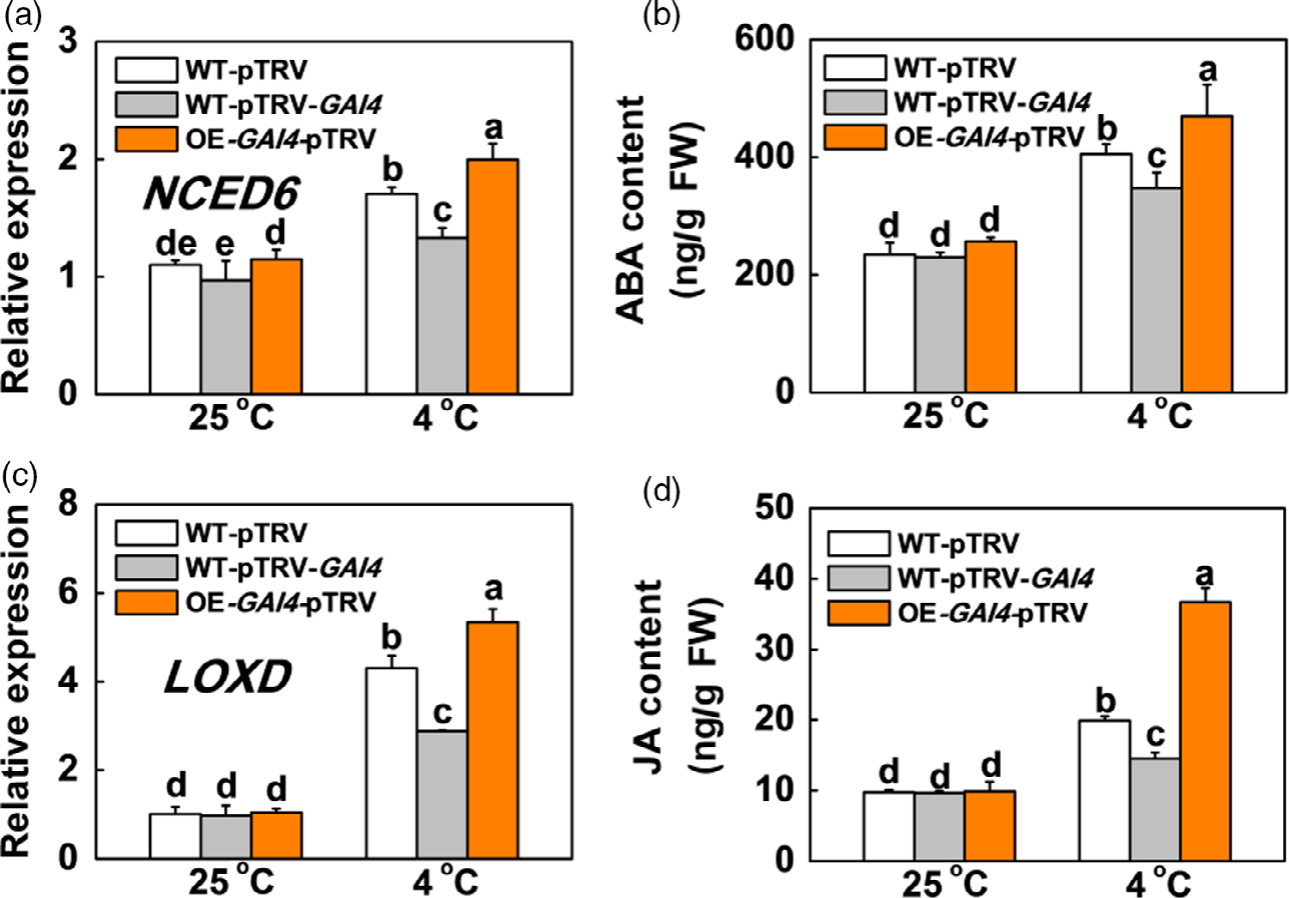
*SlGAI4* promotes expression of genes in ABA and JA pathway and their accumulation in response to cold stress. (a) and (c) Expression of *NCED6* (a) and *LOXD* (c) in tomato wild‐type (WT‐pTRV), *SlGAI4*‐silenced plants (WT‐pTRV‐*GAI4*) and *SlGAI4*‐overexpressing plants (OE‐*GAI4*‐pTRV) after exposure to 25 °C or 4 °C under low R/FR (L‐R/FR, 0.5) light conditions for 6 h. (b) and (d) Endogenous levels of ABA (b) and JA (d) biosynthesis in tomato wild‐type (WT‐pTRV), *SlGAI4*‐silenced plants (WT‐pTRV‐*GAI4*) and *SlGAI4*‐overexpressing plants (OE‐*GAI4*‐pTRV) after exposure to 25 °C or 4 °C under L‐R/FR light for 12 h. For light‐quality treatments, plants were maintained at R conditions (120 µmol m^−2^ s^−1^) and supplemented with different intensities of FR. Data are presented as the means of three biological replicates (±SD). Different letters indicate significant differences (*P* < 0.05) according to Tukey’s test.

The results described above suggested that SlPIF4 enhanced cold tolerance in tomato plants by directly activating *SlGAI4* and inducing ABA and JA biosynthesis. We then asked whether *SlGAI4* regulated the levels of ABA and JA under cold stress. To this end, we examined the ABA biosynthesis (*SlNCED6*) and signalling (*SlAREB* and *SlRD22‐like*) genes, JA biosynthesis (*SlLOXD* and *SlAOC*) and signalling genes (*SlCOI1*), and the levels of ABA and JA in WT plants (WT‐pTRV), *SlGAI4*‐silenced plants (WT‐pTRV‐*SlGAI4*) and *SlGAI4*‐overexpressing plants (OE‐*SlGAI4*‐pTRV) under cold stress. The results showed that low temperature induced the transcription of ABA‐ and JA‐related genes and their contents (Figures 7 and S11). Meanwhile, the levels of ABA, JA and their related gene expression in the *SlGAI4*‐overexpressing plants (OE‐*SlGAI4*‐pTRV) increased significantly compared with those of the WT plants (WT‐pTRV) at 4 °C. In contrast, their levels and related gene expression decreased significantly in *SlGAI4*‐silenced plants (WT‐pTRV‐*SlGAI4*) compared with the WT plants (WT‐pTRV) at 4 °C. Therefore, *SlGAI4* might be involved in cold signalling partially by positively regulating ABA and JA.

**Figure 8:**
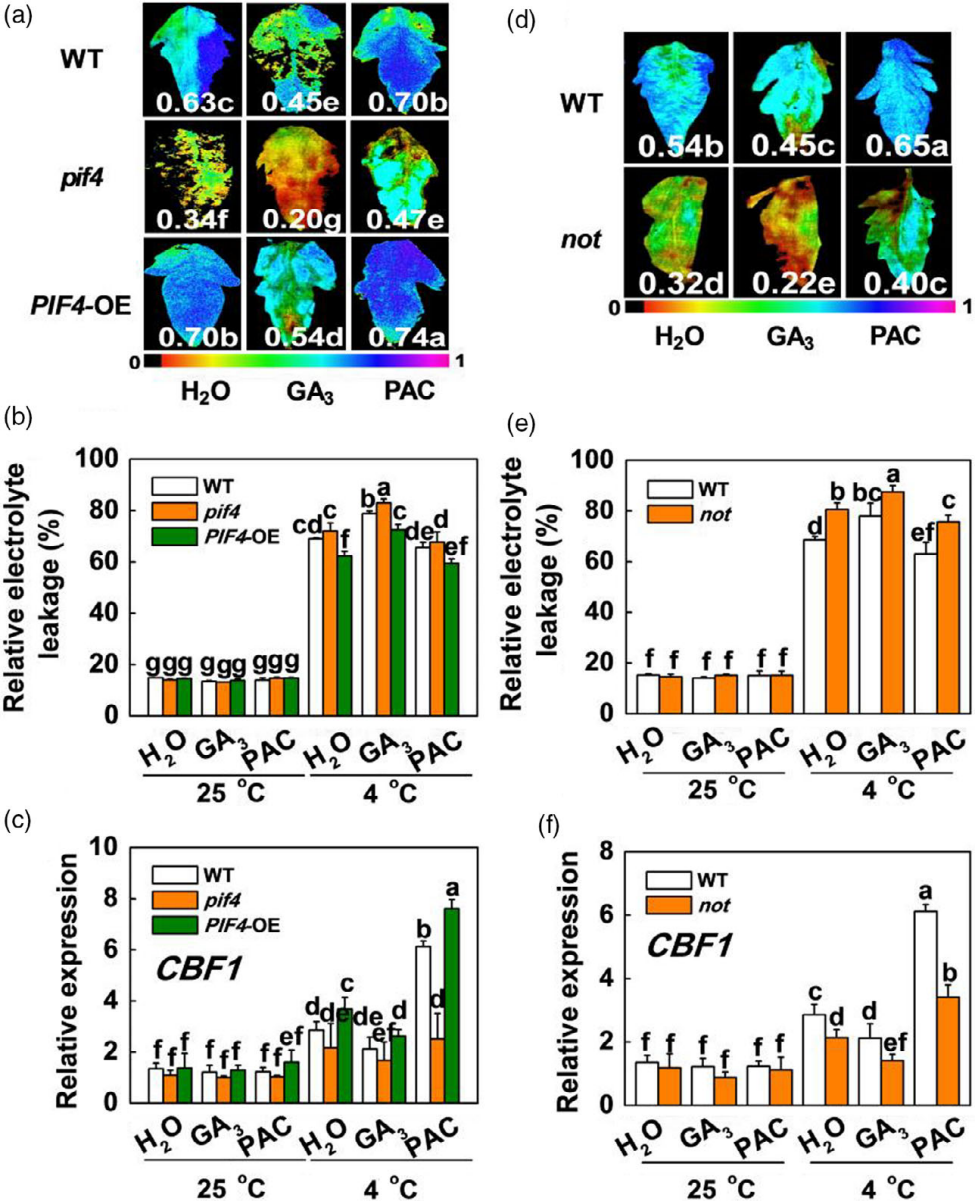
The effects of GA_3_ and PAC on cold tolerance in tomato WT, *pif4* Mutant, *SlPIF4*‐OE and *not* mutant plants. (a) Fv/Fm in tomato wild‐type (WT), *pif4* mutant (*pif4*) and *SlPIF4‐*overexpressing plants (*SlPIF4*‐OE) after exposure to 4 °C under low R/FR (L‐R/FR, 0.5) light conditions for 7 days, which pretreated with water (H_2_O), GA_3_ (50 μm) or PAC (GA biosynthesis inhibitor, 25 μm) for 12 h prior to exposure to cold conditions at 4 °C. The false‐colour code depicted at the bottom of the image ranges from 0 (black) to 1.0 (purple), representing the level of damage in the leaves. (b) and (c) REL (b) and *SlCBF1* gene expression (c) in tomato WT, *pif4* and *SlPIF4*‐OE plants after exposure to 25 °C or 4 °C under L‐R/FR light for 7 days and 6 h, respectively, which pretreated with H_2_O, GA_3_ or PAC for 12 h prior to exposure to cold conditions at 4 °C. (d) Fv/Fm in tomato wild‐type (WT) and ABA‐deficient mutant (*not*) plants after exposure to 4 °C under L‐R/FR light for 7 days, which pretreated with water (H_2_O), GA_3_ or PAC for 12 h prior to exposure to cold conditions at 4 °C. (e) and (f) REL (e) and *SlCBF1* gene expression (f) in tomato WT and *not* plants after exposure to 25 °C or 4 °C under L‐R/FR light for 7 days and 6 h, respectively, which pretreated with H_2_O, GA_3_ or PAC for 12 h prior to exposure to cold conditions at 4 °C. For light‐quality treatments, plants were maintained at R conditions (120 µmol m^−2^ s^−1^) and supplemented with different intensities of FR. Data are presented as the means of three biological replicates (±SD). Different letters indicate significant differences (*P* < 0.05) according to Tukey’s test.

To further explore the exact role of GA signalling in SlPIF4‐regulated cold tolerance in tomato plants, we tested whether the cold tolerance of the WT, *pif4* mutant and *SlPIF4*‐OE plants was affected by altered GA levels. We observed that exogenous GA_3_ significantly repressed cold tolerance, with wilted leaves, decreased Fv/Fm values and CBF‐pathway gene (*CBF1*, *CBF3*,* COR47‐like* and *COR413‐like*) transcript levels and increased REL in the *SlPIF4*‐OE and WT plants at 4 °C (Figures 8a–c and S12). In contrast, application of the GA biosynthesis inhibitor paclobutrazol (PAC) dramatically enhanced the cold tolerance of both the WT and *pif4* mutant plants, with increased Fv/Fm values and CBF‐pathway gene (*CBF1*, *CBF3*, *COR47‐like* and *COR413‐like*) transcript levels and decreased REL. These results suggest that GA signalling functions downstream of SlPIF4 in the cold response in tomato plants.

To establish whether the actions of GA and ABA in the cold response occur in a linear sequence, we analysed the changes in cold tolerance after foliar application of exogenous GA_3_ and PAC in WT and ABA‐deficient mutant (*notabilis*, *not*) tomato plants. We observed that PAC clearly enhanced the cold tolerance of WT plants, with increased Fv/Fm values and CBF‐pathway gene (*CBF1*, *CBF3*, *COR47‐like* and *COR413‐like*) transcript levels and decreased REL (Figures 8d–f and S13). However, this induction of plant cold tolerance by PAC was significantly inhibited in the *not* mutant. These results suggest that GA signalling functions upstream of ABA in the cold response in tomato plants. Collectively, these results indicate that *SlGAI4*, a key repressor of GA signalling, acts downstream of SlPIF4 to promote cold tolerance in tomato plants partially through activating ABA and JA signalling.

### *SlGAI4* negatively regulates SlPIF4 at transcriptional and post‐translational levels under cold stress

**Figure 9:**
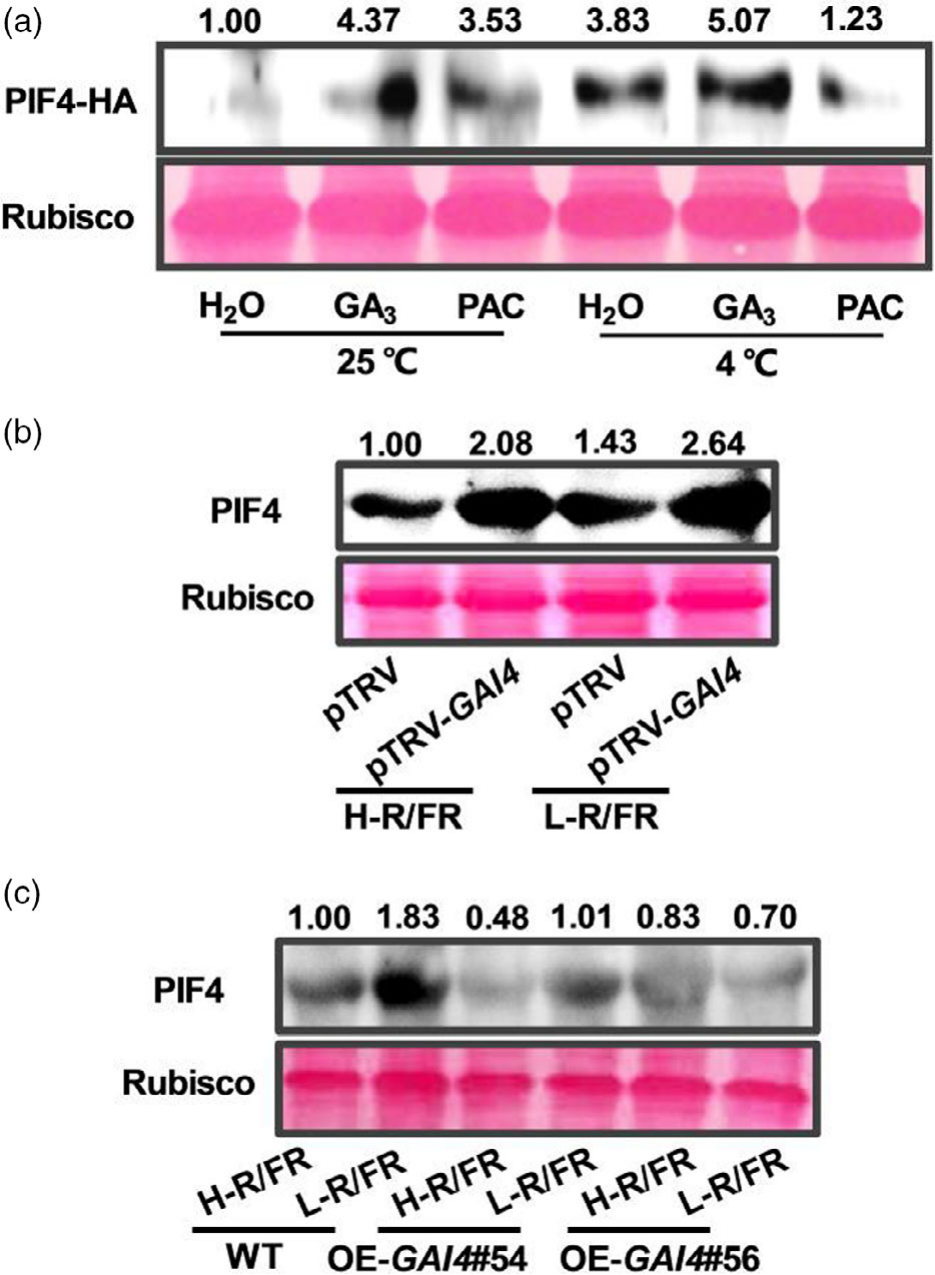
DELLAs negatively regulate SlPIF4 protein abundance at low‐temperature condition. (a) Levels of SlPIF4‐HA proteins in *35S:PIF43‐*HA tomato plants grown at 25 °C or 4 °C for 24 h. *35S:PIF43‐*HA seedlings were pretreated with water (H_2_O), GA_3_ (50 μm) or PAC (GA biosynthesis inhibitor, 25 μm) for 12 h before exposure to cold stress. (b) Levels of SlPIF4 proteins in tomato wild‐type (pTRV) and *SlGAI4*‐silenced plants (pTRV‐*GAI4*) after exposure to 4 °C under high R/FR (H‐R/FR, 2.5) light or low R/FR (L‐R/FR, 0.5) light conditions for 24 h. (c) Levels of SlPIF4 proteins in tomato wild‐type (WT) and *SlGAI4*‐overexpressing plants (OE#54, OE#56) after exposure to 4 °C under H‐R/FR or L‐R/FR conditions for 24 h. For light‐quality treatments, plants were maintained at R conditions (120 µmol m^−2^ s^−1^) and supplemented with different intensities of FR. Rubisco was used as a loading control.

It was previously reported that DELLAs interact with PIFs and block their activities by sequestering transcription factors from binding to their targets in *Arabidopsis* (Feng *et al.*, 2008; de Lucas *et al.*, 2008). To investigate whether tomato DELLAs also promoted SlPIF4 degradation under cold stress, *35S:SlPIF4*‐HA plants were treated with GA_3_ or PAC under optimal temperature conditions and cold stress, and the SlPIF4‐HA protein levels were determined. As shown in Figure 9a, the *SlPIF4*‐HA protein accumulated at increased levels at 4 °C, especially after GA_3_ application. In contrast, the *SlPIF4*‐HA protein abundance significantly decreased when PAC was applied compared with the effect of mock treatment at 4 °C (Figure 9a). These data indicate that DELLAs promote the degradation of the SlPIF4 protein. Next, we tested the transcription of *SlPIF4* and its protein abundance in *SlGAI4*‐silenced plants and *SlGAI4*‐overexpressing plants at 4 °C under H‐R/FR or L‐R/FR conditions. The results showed that *SlPIF4* transcription and its protein levels were higher in the *SlGAI4*‐silenced plants and lower in the *SlGAI4*‐overexpressing plants than in the WT plants (pTRV or WT) during cold stress (Figures 9b,c and S14). These results demonstrate that *SlGAI4* negative feedback regulates SlPIF4 at both transcriptional and post‐translational levels under cold stress.

## Discussion

**Figure 10:**
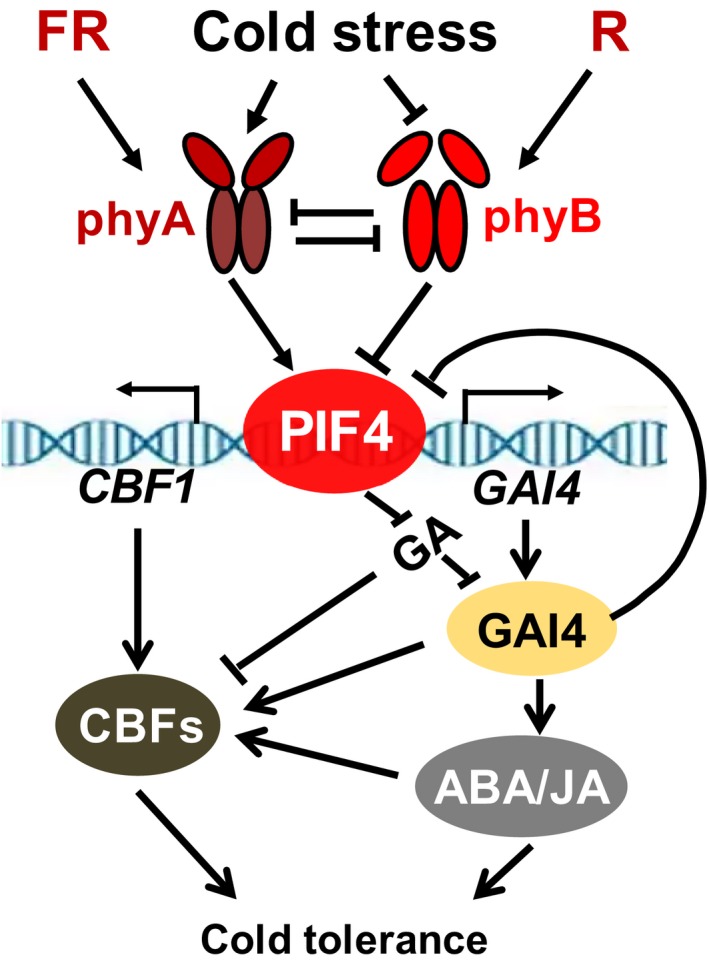
A proposed model for SlPIF4 positively regulating tomato cold tolerance by integrating light and temperature signals. Briefly, L‐R/FR and low temperature induce SlPIF4 protein accumulation via a phyA‐dependent pathway under cold stress. SlPIF4 not only directly activates *CBF* expression but also associates with the promoter of the *SlGAI4* gene and activates its transcription, promoting ABA and JA biosynthesis and *CBF* expression. Thus, SlPIF4 is a positive regulator in L‐R/FR‐induced cold tolerance in tomato. SlGAI4, a DELLA protein, acts downstream of SlPIF4 and positively regulates L‐R/FR‐induced cold tolerance. Interestingly, when large amounts of SlGAI4 protein accumulate during cold stress, it represses SlPIF4 accumulation in a negative feedback manner.

Light and temperature are arguably two of the most important environmental factors that coordinately control plant growth and survival. Light and temperature signals change with daily and seasonal fluctuations and according to latitudinal and vegetation canopy variations. For example, the R/FR ratios are reduced naturally with vegetative shading and twilight durations at northern latitudes in cool seasons (Franklin and Whitelam, 2007). We previously found that L‐R/FR ratios induced plant cold tolerance via a phyA‐dependent pathway in tomato (Wang *et al.*, 2016). Intriguingly, recent work has demonstrated that phyB acts as a thermosensor in the regulation of elongation growth in *Arabidopsis* at warm temperatures via regulation of AtPIF4 (Jung *et al.*, 2016; Legris *et al.*, 2016). AtPIF4 and AtPIF7 repress *CBF* gene transcription to decrease the unnecessary activation of the cold acclimation pathway during the warm long‐day season (Lee and Thomashow, 2012), which suggests the possibility that PIFs integrate light and low‐temperature signalling. Recently, AtPIF3 was identified as a negative regulator of freezing tolerance by directly repressing the *CBF* gene in *Arabidopsis* (Jiang *et al.*, 2017). OsPIF14 directly bound the promoter of *DREB1B* and repressed its expression in *Oryza sativa* (Cordeiro *et al.*, 2016). However, ZmPIF3 directly activated *DREB2A* gene expression in *Zea mays* under drought (Gao *et al.*, 2015), and OsPIL16 positively regulated plant cold tolerance by directly binding the promoter of the *DREB1* gene and activating its transcription in *Oryza sativa* (He *et al.*, 2016). These results indicate that PIFs are functionally diverse among different plant species. Here, we provide several lines of evidence showing that SlPIF4 is involved in L‐R/FR‐induced cold tolerance in tomato plants (Figure 10). First, FR induced SlPIF4 accumulation at low temperatures via phyA (Figure 1d–f). Second, the L‐R/FR‐induced transcript levels of *CBF1* under cold stress decreased in *pif4* mutant plants, which displayed impaired cold tolerance, but its transcript levels increased in the *SlPIF4*‐overexpression lines, which exhibited enhanced cold tolerance (Figures 2a–c and S5). Third, SlPIF4 directly bound to the G‐box of the *CBF* promoters *in vitro* and was associated with the promoters of the *CBF* genes *in vivo* (Figures 2d–g and S6). Finally, SlPIF4 promoted ABA and JA accumulation in tomato plants under cold stress (Figures 3 and S7), which were positive regulators in cold stress (Wang *et al.*, 2016), and enhanced the degradation of GA (Figures 3 and S7), which was a negative regulator in cold tolerance (Achard *et al.*, 2008; Zhou *et al.*, 2017). Collectively, our results indicate that SlPIF4 works as a positive regulator of L‐R/FR‐induced cold tolerance in tomato plants that directly activates the transcription of *CBF* genes and regulates phytohormone homeostasis.

We observed that GA_3_ significantly repressed cold tolerance in the *SlPIF4*‐OE and WT plants at 4 °C (Figures 8a–c and S12); in contrast, application of PAC dramatically enhanced the cold tolerance of both the WT and *pif4* mutant plants. These results suggest that GA signalling functions downstream of SlPIF4 in the cold response in tomato plants. Previous studies showed that PHYTOCHROME‐INTERACTING FACTOR3‐LIKE5 (PIL5) inhibited seed germination by directly binding the promoters of two *DELLA* genes (*GAI* and *RGA*) and promoting their transcription in *Arabidopsis* (Oh *et al.*, 2007). Consistent with these observations, our ChIP analyses and EMSA showed that SlPIF4 directly and specifically bound G‐box elements of the *SlGAI4* promoters *in vitro* and *in vivo* (Figure 5c,e). Dual‐luciferase assays and transcript analyses in the *pif4* mutant and *SlPIF4*‐overexpressing plants further confirmed that SlPIF4 directly activated *SlGAI4* gene expression under cold stress, especially under L‐R/FR conditions (Figures 5b,c and S8b). In addition, we provided evidence that *SlGAI4* was a positive regulator that modulates cold tolerance in tomato plants. We showed that multiple transgenic lines overexpressing *SlGAI4* displayed a decrease in REL and increases in Fv/Fm values and CBF‐pathway gene (*CBF1* and *COR413‐like*) expression under cold stress (Figures 6d,e and S10a,c). However, L‐R/FR failed to induce Fv/Fm values and CBF‐pathway gene (*CBF1* and *COR413‐like*) expression in the *SlGAI4‐*silenced plants (Figures 6b,c and S10b). Moreover, it has been reported that CBF and DELLAs positively regulate each other in response to low temperature in *Arabidopsis* (Achard *et al.*, 2008; Zhou *et al.*, 2017). Taken together, these results demonstrate that SlPIF4 also directly activated *SlGAI4*, which acts as a positive regulator in the regulation of *CBF1* gene expression and L‐R/FR‐induced cold tolerance in tomato plants during cold stress.

DELLA proteins serve as integrators of various hormonal and environmental signals (Achard *et al.*, 2006; Daviere and Achard, 2016); thus, we investigated the levels of ABA‐related genes and their contents in the *SlGAI4*‐silenced and *SlGAI4*‐overexpressing plants under cold stress. Our results indicated that *SlGAI4* positively regulated the levels of ABA‐related genes and ABA under cold stress (Figures 7a,b and S11a,b). Foliar application of exogenous GA_3_ and PAC significantly decreased and increased cold tolerance in tomato plants, respectively, while foliar application of PAC failed to fully rescue the changes in *CBF*‐related gene transcription and cold tolerance in the ABA‐deficient *not* plants (Figures 8d,f and S13). These results suggest that low temperature induces a decrease in GA levels and promotes *SlGAI4* gene expression, which may result in an increase in ABA levels in tomato plants. Consistent with this finding, DELLA proteins interact with ABI3 and ABI5 and form DELLA/ABI3/ABI5 complexes under unfavourable conditions (e.g. high or low temperature), which positively regulate ABA biosynthesis in *Arabidopsis* (Kim *et al.*, 2008; Lim *et al.*, 2013; Park *et al.*, 2011). Conversely, a previous report showed that ABA decreased GA levels by repressing GA biosynthetic genes in seeds (Seo *et al.*, 2006). Thus, it appears that ABA and GA antagonistically regulate each other. In addition, recent studies have shown that jasmonate ZIM‐domain proteins (JAZs), major repressors in JA signalling, directly target ICE1 to inhibit the activation of CBFs, while DELLAs competitively bind to JAZs to release MYC2 and activate the JA response (Hou *et al.*, 2010; Hu *et al.*, 2013; Wild *et al.*, 2012). Meanwhile, MYC2 also interacts with ICE1 to enhance *CBF* gene transcription in cold conditions (Zhao *et al.*, 2012). Here, we showed that the levels of JA‐related gene transcription and JA decreased in the *SlGAI4*‐silenced plants but increased in the *SlGAI4‐*overexpressing plants under cold stress (Figures 7c,d and S11c,d). Thus, *SlGAI4* could promote ABA and JA accumulation under cold stress (Figures 7 and S11), which would positively regulate plant cold tolerance via CBF‐dependent and CBF‐independent pathways (Eremina *et al.*, 2016; Hu *et al.*, 2013; Shinozaki and Yamaguchi‐Shinozaki, 2000; Wang *et al.*, 2016).

Previous studies demonstrated that DELLAs interact with AtPIF3 and AtPIF4 and inhibit their activities by sequestering their DNA‐recognition domains, ultimately results in the inhibition of hypocotyl elongation (Feng *et al.*, 2008; de Lucas *et al.*, 2008). Excitingly, recent studies have revealed that DELLA proteins negatively regulate PIF accumulation by inducing rapid degradation of PIFs through the 26S proteasome pathway (Li *et al.*, 2016a; Pham *et al.*, 2018). Indeed, our work showed that GA_3_ promotes SlPIF4 protein accumulation, while PAC inhibits SlPIF4 protein accumulation (Figure 9a). Furthermore, we found that the SlPIF4 protein and its transcription increased in the *SlGAI4*‐silenced plants, while its protein abundance and gene expression decreased in *SlGAI4*‐overexpressing plants compared with WT plants (Figures 9b,c and S14). These results support a role for *SlGAI4* in the negative feedback regulation of SlPIF4 at both transcriptional and post‐translational levels under cold stress. Therefore, SlPIF4 acts as a central hub that integrates light and temperature signals to orchestrate the regulation of the transcriptional network that drives multiple facets of downstream cold response. During cold stress, low temperature and FR signals induced the accumulation of SlPIF4, which directly activated *SlCBF*s and *SlGAI4* to enhance cold tolerance. *SlGAI4 ‐*induced ABA and JA signalling enhanced plant cold tolerance by regulating CBF‐dependent or CBF‐independent pathways. Since increased expression of *CBFs* and *SlGAI4* would result in plant growth cessation, *SlGAI4* forms a negative feedback loop with SlPIF4. This feedback modulation and redundant cold response pathways likely contribute to maintain appropriate levels of SlPIF4 to balance plant growth and cold tolerance.

## Conclusions

In summary, we propose a model that illustrates how light and temperature signals are integrated to regulate cold tolerance in tomato plants (Figure 10). Briefly, L‐R/FR and low temperature induce SlPIF4 accumulation via phyA, while phyB represses SlPIF4 accumulation under cold stress. SlPIF4 not only directly activates *CBF* expression but also associates with the promoter of the *SlGAI4* gene and activates its transcript, which promotes ABA and JA biosynthesis and *CBF* expression. Thus, SlPIF4 is a positive regulator in L‐R/FR‐induced cold tolerance in tomato plants. SlGAI4, a DELLA protein, acts downstream of SlPIF4 and positively regulates L‐R/FR‐induced cold tolerance. Interestingly, when large amounts of SlGAI4 protein accumulate during cold stress, it represses SlPIF4 accumulation in a negative feedback manner. In the natural environment, a decrease in temperature is often associated with longer twilight durations during autumn at northern latitudes, which are characterized by a significant drop in the R/FR ratio. Monitoring of R/FR ratio signals would provide an early warning and confer some protection to plants subject to a sudden decrease in temperature during night and seasonal variations. This study unveils a novel mechanism by which tomato plants have evolved a phytochrome‐dependent, SlPIF4‐mediated adaptation strategy by sensing and integrating environmental cues with plant hormone signals.

## Materials and methods

### Plant materials and constructs

The tomato *phyA*,* phyB1B2* and *phyAB1B2* mutants were obtained from the Tomato Genetics Resource Center (http://tgrc.ucdavis.edu). Tobacco rattle virus (TRV)‐based vectors (pTRV1/2) were used for VIGS of the *SlPIF* and *SlGAI* family genes, and VIGS was performed as described previously (Wang *et al.*, 2016). The tomato *pif4* mutant in the Ailsa Craig ecotype was obtained by using the CRISPR/Cas9 technique (Pan *et al.*, 2011; Wang *et al.*, 2018). The target sequence (AGGTCATCCAATGTGCAGCT) and its complementary sequence were annealed and inserted into the *Bbs*I site of the AtU6‐sgRNA‐AtUBQ‐Cas9 vector, and the AtU6‐sgRNA‐AtUBQ‐Cas9 cassette was inserted into the *Hind*III and *Kpn*I sites of the pCAMBIA1301 binary vector. Transgenic plants overexpressing HA‐tagged *SlPIF4* and *SlGAI4* were generated by cloning the full‐length *SlPIF4* and *SlGAI4* cDNAs into the pFGC1008‐HA vector, which contains a CaMV 35S promoter, transforming the vectors into *Agrobacterium tumefaciens* strain EHA105, and then introducing them into tomato seeds of ecotype Ailsa Craig via a previously described method (Fillatti *et al.*, 1987). All primers used for plasmid construction are listed in Table S1. Two independent homozygous lines of the F2 generation in *SlPIF4‐* and *SlGAI4‐*overexpressing plants and two independent *pif4* lines, which were mutated at the first base of the protospacer adjacent motif (PAM) to stop translation immediately, were used for the study. In tomato *pif4#3* and *pif4#10* mutants, there was a single nucleotide (T) insertion at 3‐bp upstream of the PAM sites and 2‐bp (GC) deletion at 4‐bp upstream of the PAM sites in the sgRNA, respectively, as showed in Figure S3a–c. The growth conditions for the tomato mutants and overexpressing seedlings were as follows: temperature of 25 °C/20 °C (day/night), 12‐h light/dark cycles and photosynthetic photon flux density (PPFD) of 600 µmol m^−2^ s^−1^. VIGS plants were grown at 21 °C/20 °C under 12‐h light/dark cycles.

### Cold and light treatments

Plants at the 4‐leaf stage were used for all experiments, which were carried out in controlled‐environment growth chambers (Zhejiang Qiushi Artificial Environment Co., Ltd, China). Plants were grown under dark (D) or white light (WL), red light (R) and FR light conditions with an aerial temperature of 25 °C or 4 °C for 12‐h cold treatment. The light intensities of WL, R and FR were 120 µmol m^−2^ s^−1^. For the different R/FR ratio (high R/FR ratios, 2.5, or low R/FR ratios, 0.5) treatments, R (λ_max_ = 660 nm, Philips, Netherlands) light intensity was maintained at 120 µmol m^−2^ s^−1^, and FR (λ_max_ = 735 nm, Philips, Netherlands) was added. The R/FR ratio was calculated as the quantum flux density from 655 to 665 nm divided by the quantum flux density from 730 to 740 nm. Plants remained under 12‐h light/dark cycles while exposed to the cold treatment. The cold treatment at 4 °C lasted for 7 days, unless stated otherwise in the text.

### GA_3_ and PAC treatment

To unveil the relationship between GA and ABA in cold tolerance, 50 μm GA_3_ or 25 μm PAC (GA biosynthesis inhibitor) was applied on WT and *not* plants 12 h prior to exposure to cold conditions at 4 °C under low R/FR (L‐R/FR, 0.5) light conditions for 7 days. To determine the effect of DELLAs on SlPIF4 in cold tolerance, WT, *pif4* and *SlPIF4*‐OE plants were pretreated with 50 μm GA_3_ or 25 μm PAC prior to cold treatment at 4 °C under L‐R/FR light for 7 days. The GA_3_ (Sigma‐Aldrich, St. Louis, MO, USA) and PAC (Sigma‐Aldrich) solutions were prepared by dissolving the solutes in ethanol followed by dilution with distilled water (ethanol : water [v/v] = 1 : 10 000), respectively. The cold tolerance of tomato plants was analysed after foliar application with 20 mL solution or water on each plant.

### Cold tolerance and hypocotyl length assays

The REL, indicating the membrane permeability, was measured as described previously (Cao *et al.*, 2007). The maximum quantum yield of PSII (Fv/Fm) in the leaves was assayed by using the Imaging‐PAM set‐up (IMAG‐MAXI; Heinz Walz, Germany), as previously described (Wang *et al.*, 2018). Hypocotyl length was measured after the germination seeding under white light (12‐h light/12‐h dark) or dark (24‐h dark) conditions for 7 days.

### Determination of ABA, JA and GA levels

Endogenous ABA and JA were extracted from tomato leaves and determined by LC/MS‐MS on an Agilent 1290 Infinity HPLC system coupled to an Agilent 6460 Triple Quad LC‐MS device (Agilent Technologies, Amstelveen, the Netherlands), as described previously (Wang *et al.*, 2016). GA was extracted from 1‐g samples of tomato leaves and quantified by a derivation approach coupled with nano‐LC‐ESI‐Q‐TOF‐MS analysis as described previously (Chen *et al*., 2012; Wang *et al.*, 2019). For the determination of GA levels, D_2_‐GA_1_, D_2_‐GA_3_, D_2_‐GA_4_, D_2_‐GA_9_, D_2_‐GA_19_ and D_2_‐GA_20_ were added to the extraction solution as internal standards.

### Phylogenetic analysis

The amino acid sequences of the eight *Arabidopsis thaliana* canonical PIF proteins (Leivar and Quail, 2011) were used as queries to perform a BLAST search against Sol Genomics databases (https://solgenomics.net/). Sequence alignment and phylogenetic tree construction were determined with MEGA 6 software using the corrected Nei–Gojobori method. A consensus neighbour‐joining tree was obtained from 1000 bootstrap replicates of aligned sequences. The percentage at the branch points represents the posterior probabilities of amino acid sequences.

### Isolation of RNA and qRT‐PCR

Total RNA was isolated using an RNAprep Pure Plant Kit (Tiangen Biotech Co., Ltd., Beijing, China) from tomato leaves under different conditions as indicated in the figure legend. The extracted RNA was reverse‐transcribed using a ReverTra Ace qPCR RT Kit with an enzyme for genomic DNA removal (Toyobo, Osaka, Japan). qRT‐PCR was performed with SYBR Green PCR Master Mix (Takara, Japan) using a LightCycler 480 II detection system (Roche, Germany). The PCR procedure was described previously (Wang *et al.*, 2018). The expression levels were normalized to the expression of tomato *ACTIN2* gene, which was stably expressed in tomato plants under cold and light stress combined conditions by geNorm algorithm (Livak and Schmittgen, 2001; Løvdal and Lillo, 2009). Primers are listed in Table S2.

### Immunoblotting assays

Total proteins were extracted from tomato leaves by homogenization in extraction buffer as described previously (Wang *et al.*, 2019). Protein concentrations were measured using Coomassie stain (Bradford, 1976). Equal amounts of total proteins from each sample were subjected to 15% SDS‐PAGE and electrotransferred to nitrocellulose membranes (Bio‐Rad, Hercules, CA). The proteins were blotted with antibodies against PIF4 (AS163955; Agrisera) or anti‐HA (Cat. No. 26183; Pierce) and subsequently with horseradish‐peroxidase‐conjugated secondary antibody (anti‐goat, Invitrogen, Sweden). The signals were visualized with enhanced chemical luminescence (ECL).

### Electrophoretic mobility shift assay

The pET‐32a‐His‐SlPIF4 vector was generated using the full‐length coding region of SlPIF4 with the primers listed in Table S1. His‐tagged SlPIF4 protein was expressed in *Escherichia coli* strain BL21 (DE3) and purified with the manufacturer’s instructions of the Novagen pET purification system. EMSA was performed using biotin‐labelled probes and the LightShift Chemiluminescent EMSA Kit (Cat. no. 20148; Thermo Fisher Scientific). The SlPIF4 proteins and biotin‐labelled probe were incubated together in binding buffer for 20 min at room temperature, the reaction mixture was resolved by 6% non‐denaturing polyacrylamide gel in Tris–glycine buffer and electrophoresed at 100 V, then transferred to a positive nylon membrane, and subjected to UV cross‐linking. Finally, the protein‐DNA signals were detected by chemiluminescence according to the instructions of the LightShift Chemiluminescent EMSA Kit. The sequences of the biotin‐labelled are shown in Table S3.

### Chromatin immunoprecipitation assay

ChIP‐PCR assays were performed following the manufacturer’s instructions for the EpiQuik™ Plant ChIP Kit (Cat. No. P‐2014; EpiGentek) as previously described (Wang *et al.*, 2018). Approximately 1 g of leaf tissue was harvested from *SlPIF4*‐OE and WT plants, which were grown at 4 °C under L‐R/FR conditions for 1 day and were treated with formaldehyde to cross‐link the protein‐DNA complexes. The chromatin complexes containing SlPIF4‐3HA fusion protein were immunoprecipitated with an anti‐HA antibody (Cat. No. 26183; Pierce) and Protein A Agarose beads (GE). Goat anti‐mouse IgG (Cat. No. AP124P; Millipore) was used as a negative control. The immunoprecipitated DNA was analysed by qPCR using gene‐specific primers which are listed in Table S4.

### Dual‐luciferase assays

SlPIF4 full‐length and the *GAI4* promoter fragment were cloned into the pGreenII 0029 62‐SK and pGreenII 0800‐LUC vectors, respectively (Figure S8b). The recombinant vectors were transformed into *Agrobacterium* strain GV3101. The pGreenII 0029 62‐SK empty vector was used as a negative control, and the 35S promoter‐driven Renilla luciferase was used as an internal control. Different combinations of strains were injected into the back of tobacco leaves. After infiltration, the tobacco plants were grown at 25 °C for 24 h, and then, one group of these plants was transferred to 4 °C for 24 h before taking samples. The tobacco leaves were ground, and the extraction solutions were incubated in buffer at a low temperature. LUC/REN was detected with an enzyme standard instrument (SpectraMax iD5, Tecan, Basel, Switzerland) by using a Modulus Luminometer (Promega, Madison, WI) as previously described (Yin *et al.*, 2016).

### Statistical analyses

Three biological replicates for each treatment were used with at least 6 plants for each replicate. The experiments were independently performed three times. To determine statistical significance, we employed Tukey’s least significant difference (LSD) test. The difference was considered significant at *P* < 0.05 and indicated by different letters.

## Accession numbers

Sequence data from this article can be found in the Sol Genomics databases (https://solgenomics.net/) under the accession numbers listed in Tables S2, S3 and S4.

## Conflict of interest

The authors declare no conflict of interests.

## Author contributions

Y.Z. and J.Y. designed the research, F.W., X.C., D.S., X.J. and L.W. performed the experiments. F.W. and X.C. analysed the data. F.W. and Y.Z. wrote the paper.

## Supporting information


**Figure S1** Phylogenetic analysis of tomato PIF family genes (SlPIFs).
**Figure S2** Low R/FR enhances the cold tolerance in tomato plants.
**Figure S3** Tomato pif4 mutant and SlPIF4‐overexpressing plants.
**Figure S4** SlPIF4 has no effect on the hypocotyl length in tomato plants.
**Figure S5** Phenotypes (a) and expression of COR413‐like gene (b) in tomato WT, pif4 mutants and SlPIF4‐OE plants after exposure to 25 °C or 4 °C for 7 days or 6 h, respectively, under high R/FR or low R/FR light conditions.
**Figure S6** ChIP‐qPCR assay shows the relative amount of SlCBF2 and SlCBF3 fragments in 35S: SlPIF4‐HA and wild‐type tomato plants.
**Figure S7** SlPIF4 positively regulates expression of ABA and JA signalling genes in response to cold stress.
**Figure S8** Phylogenetic analysis of tomato GAI family genes (SlGAIs) and schematic diagram showing vectors construction in dual‐luciferase assays.
**Figure S9** Expression of SlGAI4 gene in wild‐type (WT/pTRV), SlGAI4‐silenced plants (pTRV‐GAI4) and SlGAI4‐overexpressing plants (OE#54, OE#56).
**Figure S10** Phenotypes (a) and expression of COR413‐like gene in tomato SlGAI4‐silenced plants (pTRV‐GAI4; b) and SlGAI4‐overexpressing plants (OE#54, OE#56; c) after exposure to 25 °C or 4 °C for 7 days or 6 h, respectively, under high R/FR or low R/FR light conditions.
**Figure S11** SlGAI4 positively regulates expression of ABA and JA signalling genes in response to cold stress.
**Figure S12** The effects of GA3 and PAC on cold tolerance in tomato WT, pif4 mutant and SlPIF4‐OE plants.
**Figure S13** The effects of GA3 and PAC on cold tolerance in tomato WT and not plants.
**Figure S14** Expression of SlPIF4 in tomato SlGAI4‐silenced plants (a) and SlGAI4‐overexpressing plants (b) after exposure to 25 °C or 4 °C under H‐R/FR or L‐R/FR conditions for 6 h.
**Table S1** PCR primer sequences used for vector construction
**Table S2** List of primer sequences used for qRT‐PCR analysis
**Table S3** Probes used in the electrophoretic mobility shift assays (EMSA)
**Table S4** Primers used for ChIP‐qPCR assays

## References

[pbi13272-bib-0001] Achard, P. , Cheng, H. , De Grauwe, L. , Decat, J. , Schoutteten, H. , Moritz, T. , Van Der Straeten, D. *et al.* (2006) Integration of plant responses to environmentally activated phytohormonal signals. Science, 311, 91–94. 16400150 10.1126/science.1118642

[pbi13272-bib-0002] Achard, P. , Gong, F. , Cheminant, S. , Alioua, M. , Hedden, P. and Genschik, P. (2008) The cold‐inducible CBF1 factor‐dependent signaling pathway modulates the accumulation of the growth‐repressing DELLA proteins via its effect on gibberellin metabolism. Plant Cell, 20, 2117–2129. 18757556 10.1105/tpc.108.058941 PMC2553604

[pbi13272-bib-0003] Bradford, M.M. (1976) A rapid and sensitive method for the quantitation of microgram quantities of protein utilizing the principle of protein‐dye binding. Anal. Biochem. 72, 248–254. 942051 10.1016/0003-2697(76)90527-3

[pbi13272-bib-0004] Cao, W.H. , Liu, J. , He, X.J. , Mu, R.L. , Zhou, H.L. , Chen, S.Y. and Zhang, J.S. (2007) Modulation of ethylene responses affects plant salt‐stress responses. Plant Physiol. 143, 707–719. 17189334 10.1104/pp.106.094292 PMC1803741

[pbi13272-bib-0005] Casal, J.J. and Qüesta, J.I. (2018) Light and temperature cues: multitasking receptors and transcriptional integrators. New Phytol. 217, 1029–1034. 29139132 10.1111/nph.14890

[pbi13272-bib-0006] Catalá, R. , Medina, J. and Salinas, J. (2011) Integration of low temperature and light signaling during cold acclimation response in Arabidopsis. Proc. Natl. Acad. Sci. USA, 108, 16475–16480. 21930922 10.1073/pnas.1107161108 PMC3182711

[pbi13272-bib-0007] Chen, M.L. , Fu, X.M. , Liu, J.Q. , Ye, T.T. , Hou, S.Y. , Huang, Y.Q. , Yuan, B.F. , *et al* (2012) Highly sensitive and quantitative profiling of axidic phytohormones using derivatization approach coupled with nano-LC-ESI-Q-TOF-MS analysis. J. Chromatogr. B-Analyt. Technol. Biomed. Life Sci. 905, 67–74. 22917596 10.1016/j.jchromb.2012.08.005

[pbi13272-bib-0008] Chinnusamy, V. , Zhu, J. and Zhu, J.K. (2007) Cold stress regulation of gene expression in plants. Trends Plant Sci. 12, 444–451. 17855156 10.1016/j.tplants.2007.07.002

[pbi13272-bib-0009] Cordeiro, A.M. , Figueiredo, D.D. , Tepperman, J. , Borba, A.R. , Lourenco, T. , Abreu, I.A. , Ouwerkerk, P.B. *et al.* (2016) Rice phytochrome‐interacting factor protein OsPIF14 represses *OsDREB1B* gene expression through an extended N‐box and interacts preferentially with the active form of phytochrome B. Biochim. Biophys. Acta, 1859, 393–404. 26732823 10.1016/j.bbagrm.2015.12.008 PMC4824199

[pbi13272-bib-0010] Daviere, J.M. and Achard, P. (2016) A pivotal role of DELLAs in regulating multiple hormone signals. Mol. Plant, 9, 10–20. 26415696 10.1016/j.molp.2015.09.011

[pbi13272-bib-0011] Delker, C. , Sonntag, L. , James, G.V. , Janitza, P. , Ibañez, C. , Ziermann, H. , Peterson, T. *et al.* (2014) The DET1‐COP1‐HY5 pathway constitutes a multipurpose signaling module regulating plant photomorphogenesis and thermomorphogenesis. Cell Rep. 9, 1983–1989. 25533339 10.1016/j.celrep.2014.11.043

[pbi13272-bib-0012] Dong, M.A. , Farre, E.M. and Thomashow, M.F. (2011) Circadian clock‐associated 1 and late elongated hypocotyl regulate expression of the C‐repeat binding factor (CBF) pathway in Arabidopsis. Proc. Natl. Acad. Sci. USA, 108, 7241–7246. 21471455 10.1073/pnas.1103741108 PMC3084081

[pbi13272-bib-0013] Eremina, M. , Rozhon, W. and Poppenberger, B. (2016) Hormonal control of cold stress responses in plants. Cell. Mol. Life Sci. 73, 797–810. 26598281 10.1007/s00018-015-2089-6 PMC11108489

[pbi13272-bib-0014] Feng, S. , Martinez, C. , Gusmaroli, G. , Wang, Y. , Zhou, J. , Wang, F. , Chen, L. , *et al.* (2008) Coordinated regulation of *Arabidopsis thaliana* development by light and gibberellins. Nature, 451, 475–479. 18216856 10.1038/nature06448 PMC2562044

[pbi13272-bib-0015] Fillatti, J.J. , Kiser, J. , Rose, R. and Comai, L. (1987) Efficient transfer of a glyphosate tolerance gene into tomato using a binary *Agrobacterium tumefaciens* vector. Nat. Bio‐Technol. 5, 726–730.

[pbi13272-bib-0016] Franklin, K.A. (2009) Light and temperature signal crosstalk in plant development. Curr. Opin. Plant Biol. 12, 63–68. 18951837 10.1016/j.pbi.2008.09.007

[pbi13272-bib-0017] Franklin, K.A. and Whitelam, G.C. (2007) Light‐quality regulation of freezing tolerance in *Arabidopsis thaliana* . Nat. Genet. 39, 1410–1413. 17965713 10.1038/ng.2007.3

[pbi13272-bib-0018] Franklin, K.A. , Toledo‐Ortiz, G. , Pyott, D.E. and Halliday, K.J. (2014) Interaction of light and temperature signaling. J. Exp. Bot. 65, 2859–2871. 24569036 10.1093/jxb/eru059

[pbi13272-bib-0019] Gangappa, S.N. and Kumar, S.V. (2017) DET1 and HY5 control PIF4‐mediated thermosensory elongation growth through distinct mechanisms. Cell Rep. 18, 344–351. 28076780 10.1016/j.celrep.2016.12.046 PMC5263232

[pbi13272-bib-0020] Gao, Y. , Jiang, W. , Dai, Y. , Xiao, N. , Zhang, C. , Li, H. , Lu, Y. *et al.* (2015) A maize phytochrome‐interacting factor 3 improves drought and salt stress tolerance in rice. Plant Mol. Biol. 87, 413–428. 25636202 10.1007/s11103-015-0288-z

[pbi13272-bib-0021] Gilmour, S.J. , Sebolt, A.M. , Salazar, M.P. , Everard, J.D. and Thomashow, M.F. (2000) Overexpression of the Arabidopsis *CBF3* transcriptional activator mimics multiple biochemical changes associated with cold acclimation. Plant Physiol. 124, 1854–1865. 11115899 10.1104/pp.124.4.1854 PMC59880

[pbi13272-bib-0022] He, Y.N. , Li, Y.P. , Cui, L.X. , Xie, L.X. , Zheng, C.K. , Zhou, G.H. , Zhou, J.J. *et al.* (2016) Phytochrome B negatively affects cold tolerance by regulating *OsDREB1* gene expression through phytochrome interacting factor‐like protein OsPIL16 in rice. Front. Plant Sci. 7, 1963. 28083003 10.3389/fpls.2016.01963 PMC5183628

[pbi13272-bib-0023] Hou, X.L. , Lee, L.Y. , Xia, K. , Yan, Y. and Yu, H. (2010) DELLAs modulate jasmonate signaling via competitive binding to JAZs. Dev. Cell, 19, 884–894. 21145503 10.1016/j.devcel.2010.10.024

[pbi13272-bib-0024] Hu, Y. , Jiang, L. , Wang, F. and Yu, D. (2013) Jasmonate regulates the INDUCER of CBF EXPRESSION–C‐REPEAT BINDING FACTOR/DRE BINDING FACTOR1 cascade and freezing tolerance in *Arabidopsis* . Plant Cell, 25, 2907–2924. 23933884 10.1105/tpc.113.112631 PMC3784588

[pbi13272-bib-0025] Jaglo‐Ottosen, K.R. , Gilmour, S.J. , Zarka, D.G. , Schabenberger, O. and Thomashow, M.F. (1998) *Arabidopsis CBF1* overexpression induces *COR* genes and enhances freezing tolerance. Science, 280, 104–106. 9525853 10.1126/science.280.5360.104

[pbi13272-bib-0026] Jia, Y. , Ding, Y. , Shi, Y. , Zhang, X. , Gong, Z. and Yang, S. (2016) The *cbfs* triple mutants reveal the essential functions of CBFs in cold acclimation and allow the definition of CBF regulons in *Arabidopsis* . New Phytol. 212, 345–353. 27353960 10.1111/nph.14088

[pbi13272-bib-0027] Jiang, B. , Shi, Y. , Zhang, X. , Xin, X. , Qi, L. , Guo, H. , Li, J. *et al.* (2017) PIF3 is a negative regulator of the CBF pathway and freezing tolerance in *Arabidopsis* . Proc. Natl. Acad. Sci. USA, 114, E6695–E6702. 28739888 10.1073/pnas.1706226114 PMC5559041

[pbi13272-bib-0028] Jung, J.H. , Domijan, M. , Klose, C. , Biswas, S. , Ezer, D. , Gao, M. , Khattak, A.K. *et al.* (2016) Phytochromes function as thermosensors in *Arabidopsis* . Science, 354, 886–889. 27789797 10.1126/science.aaf6005

[pbi13272-bib-0029] Kim, H.J. , Kim, Y.K. , Park, J.Y. and Kim, J. (2002) Light signalling mediated by phytochrome plays an important role in cold‐induced gene expression through the C‐repeat/dehydration responsive element (C/DRE) in *Arabidopsis thaliana* . Plant J. 29, 693–704. 12148528 10.1046/j.1365-313x.2002.01249.x

[pbi13272-bib-0030] Kim, J. , Yi, H. , Choi, G. , Shin, B. , Song, P.S. and Choi, G. (2003) Functional characterization of phytochrome interacting factor 3 in phytochrome‐mediated light signal transduction. Plant Cell, 15, 2399–2407. 14508006 10.1105/tpc.014498 PMC197304

[pbi13272-bib-0031] Kim, D.H. , Yamaguchi, S. , Lim, S. , Oh, E. , Park, J. , Hanada, A. , Kamiya, Y. *et al.* (2008) SOMNUS, a CCCH‐type zinc finger protein in *Arabidopsis*, negatively regulates light‐dependent seed germination downstream of PIL5. Plant Cell, 20, 1260–1277. 18487351 10.1105/tpc.108.058859 PMC2438461

[pbi13272-bib-0032] Kumar, S.V. , Lucyshyn, D. , Jaeger, K.E. , Alós, E. , Alvey, E. , Harberd, N.P. and Wigge, P.A. (2012) Transcription factor PIF4 controls the thermosensory activation of flowering. Nature, 484, 242–245. 22437497 10.1038/nature10928 PMC4972390

[pbi13272-bib-0033] Lee, C.M. and Thomashow, M.F. (2012) Photoperiodic regulation of the C‐repeat binding factor (CBF) cold acclimation pathway and freezing tolerance in *Arabidopsis thaliana* . Proc. Natl. Acad. Sci. USA, 109, 15054–15059. 22927419 10.1073/pnas.1211295109 PMC3443188

[pbi13272-bib-0034] Legris, M. , Klose, C. , Burgie, E.S. , Rojas, C.C. , Neme, M. , Hiltbrunner, A. , Wigge, P.A. *et al.* (2016) Phytochrome B integrates light and temperature signals in *Arabidopsis* . Science, 354, 897–900. 27789798 10.1126/science.aaf5656

[pbi13272-bib-0035] Legris, M. , Nieto, C. , Sellaro, R. , Prat, S. and Casal, J.J. (2017) Perception and signalling of light and temperature cues in plants. Plant J. 90, 683–697. 28008680 10.1111/tpj.13467

[pbi13272-bib-0036] Leivar, P. and Monte, E. (2014) PIFs: systems integrators in plant development. Plant Cell, 26, 56–78. 24481072 10.1105/tpc.113.120857 PMC3963594

[pbi13272-bib-0037] Leivar, P. and Quail, P.H. (2011) PIFs: pivotal components in a cellular signaling hub. Trends Plant Sci. 16, 19–28. 20833098 10.1016/j.tplants.2010.08.003 PMC3019249

[pbi13272-bib-0038] Li, K. , Yu, R. , Fan, L.M. , Wei, N. , Chen, H. and Deng, X.W. (2016a) DELLA‐mediated PIF degradation contributes to coordination of light and gibberellin signalling in *Arabidopsis* . Nat. Commun. 7, 11868. 27282989 10.1038/ncomms11868 PMC4906400

[pbi13272-bib-0039] Li, X. , Ma, D.B. , Lu, S.X. , Hu, X. , Huang, R.F. , Liang, T. , Xu, T.D. *et al.* (2016b) Blue light‐ and low temperature‐regulated *COR27* and *COR28* play roles in the *Arabidopsis* circadian clock. Plant Cell, 28, 2755–2769. 27837007 10.1105/tpc.16.00354 PMC5155342

[pbi13272-bib-0040] Li, H. , Ye, K.Y. , Shi, Y.T. , Cheng, J.K. , Zhang, X.Y. and Yang, S.H. (2017) BZR1 positively regulates freezing tolerance via CBF‐dependent and CBF‐independent pathways in *Arabidopsis* . Mol. Plant, 10, 545–559. 28089951 10.1016/j.molp.2017.01.004

[pbi13272-bib-0041] Lim, S. , Park, J. , Lee, N. , Jeong, J. , Toh, S. , Watanabe, A. , Kim, J. *et al.* (2013) ABA‐insensitive 3, ABA‐insensitive 5, and DELLAs interact to activate the expression of *SOMNUS* and other high‐temperature‐inducible genes in imbibed seeds in *Arabidopsis* . Plant Cell, 25, 4863–4878. 24326588 10.1105/tpc.113.118604 PMC3903992

[pbi13272-bib-0042] Livak, K.J. and Schmittgen, T.D. (2001) Analysis of relative gene expression data using real‐time quantitative PCR and the 2^−^ ^ΔΔΔ^ *^C^* ^T^ method. Methods, 25, 402–408. 11846609 10.1006/meth.2001.1262

[pbi13272-bib-0043] Lorrain, S. , Allen, T. , Duek, P.D. , Whitelam, G.C. and Fankhauser, C. (2008) Phytochrome-mediated inhibition of shade avoidance involves degradation of growth-promoting bHLH transcription factors. Plant J. 53, 312–323. 18047474 10.1111/j.1365-313X.2007.03341.x

[pbi13272-bib-0044] Løvdal, T. and Lillo, C. (2009) Reference gene selection for quantitative real‐time PCR normalization in tomato subjected to nitrogen, cold, and light stress. Anal. Biochem. 387, 238–242. 19454243 10.1016/j.ab.2009.01.024

[pbi13272-bib-0045] de Lucas, M. , Davière, J.M. , Rodríguez‐Falcón, M. , Pontin, M. , Iglesias‐Pedraz, J.M. , Lorrain, S. , Fankhauser, C. *et al.* (2008) A molecular framework for light and gibberellin control of cell elongation. Nature, 451, 480–484. 18216857 10.1038/nature06520

[pbi13272-bib-0046] Oh, E. , Yamaguchi, S. , Hu, J.H. , Yusuke, J. , Jung, B. , Paik, I. , Lee, H.S. *et al.* (2007) PIL5, a phytochrome‐interacting bHLH protein, regulates gibberellin responsiveness by binding directly to the GAI and RGA promoters in *Arabidopsis* seeds. Plant Cell, 19, 1192–1208. 17449805 10.1105/tpc.107.050153 PMC1913757

[pbi13272-bib-0047] Park, J. , Lee, N. , Kim, W. , Lim, S. and Choi, G. (2011) ABI3 and PIL5 collaboratively activate the expression of *SOMNUS* by directly binding to its promoter in imbibed *Arabidopsis* seeds. Plant Cell, 23, 1404–1415. 21467583 10.1105/tpc.110.080721 PMC3101561

[pbi13272-bib-0048] Pham, V.V. , Kathare, P.K. and Huq, E. (2018) Phytochromes and phytochrome interacting factors. Plant Physiol. 176, 1025–1038. 29138351 10.1104/pp.17.01384 PMC5813575

[pbi13272-bib-0049] Ross, M.S. , Flanagan, L.B. and Laroi, G.H. (1986) Seasonal and successional changes in light quality and quantity in the understory of boreal forest ecosystems. Can. J. Bot. 64, 2792–2799.

[pbi13272-bib-0050] Sakuraba, Y. , Jeong, J. , Kang, M.Y. , Kim, J. , Paek, N.C. and Choi, G. (2014) Phytochrome‐interacting transcription factors PIF4 and PIF5 induce leaf senescence in *Arabidopsis* . Nat. Commun. 5, 4636. 25119965 10.1038/ncomms5636

[pbi13272-bib-0051] Seo, M. , Hanada, A. , Kuwahara, A. , Endo, A. , Okamoto, M. , Yamauchi, Y. , North, H. *et al.* (2006) Regulation of hormone metabolism in Arabidopsis seeds: phytochrome regulation of abscisic acid metabolism and abscisic acid regulation of gibberellin metabolism. Plant J. 48, 354–366. 17010113 10.1111/j.1365-313X.2006.02881.x

[pbi13272-bib-0052] Shinozaki, K. and Yamaguchi‐Shinozaki, K. (2000) Molecular responses to dehydration and low temperature: differences and crosstalk between two stress signaling pathways. Curr. Opin. Plant Biol. 3, 217–223. 10837265

[pbi13272-bib-0053] Thomashow, M.F. (1999) PLANT COLD ACCLIMATION: freezing tolerance genes and regulatory mechanisms. Annu. Rev. Plant Physiol. Plant Mol. Biol. 50, 571–599. 15012220 10.1146/annurev.arplant.50.1.571

[pbi13272-bib-0054] Toledo‐Ortiz, G. , Johansson, H. , Lee, K.P. , Bou‐Torrent, J. , Stewart, K. , Steel, G. , Rodríguez‐Concepción, M. *et al.* (2014) The HY5‐PIF regulatory module coordinates light and temperature control of photosynthetic gene transcription. PLoS Genet. 10, e1004416. 24922306 10.1371/journal.pgen.1004416 PMC4055456

[pbi13272-bib-0055] Ueguchi‐Tanaka, M. , Nakajima, M. , Motoyuki, A. and Matsuoka, M. (2007) Gibberellin receptor and its role in gibberellin signaling in plants. Annu. Rev. Plant Biol. 58, 183–198. 17472566 10.1146/annurev.arplant.58.032806.103830

[pbi13272-bib-0056] Wang, F. , Guo, Z.X. , Li, H.Z. , Wang, M.M. , Onac, E. , Zhou, J. , Xia, X.J. *et al.* (2016) Phytochrome A and B function antagonistically to regulate cold tolerance via abscisic acid‐dependent jasmonate signaling. Plant Physiol. 170, 459–471. 26527654 10.1104/pp.15.01171 PMC4704577

[pbi13272-bib-0057] Wang, F. , Wu, N. , Zhang, L.Y. , Ahammed, G.J. , Chen, X.X. , Xiang, X. , Zhou, J. *et al.* (2018) Light signaling‐dependent regulation of photoinhibition and photoprotection in tomato. Plant Physiol. 176, 1311–1326. 29146776 10.1104/pp.17.01143 PMC5813521

[pbi13272-bib-0058] Wang, F. , Zhang, L.Y. , Chen, X.X. , Wu, X.D. , Xiang, X. , Zhou, J. , Xia, X.J. *et al.* (2019) SlHY5 integrates temperature, light and hormone signaling to balance plant growth and cold tolerance. Plant Physiol. 179, 749–760. 30563923 10.1104/pp.18.01140 PMC6426432

[pbi13272-bib-0059] Wild, M. , Daviere, J.M. , Cheminant, S. , Regnault, T. , Baumberger, N. , Heintz, D. , Baltz, R. *et al.* (2012) The *Arabidopsis* DELLA *RGA‐LIKE3* is a direct target of MYC2 and modulates jasmonate signaling responses. Plant Cell, 24, 3307–3319. 22892320 10.1105/tpc.112.101428 PMC3462633

[pbi13272-bib-0060] Yin, X.R. , Xie, X.L. , Xia, X.J. , Yu, J.Q. , Ferguson, I.B. , Giovannoni, J.J. and Chen, K.S. (2016) Involvement of an ethylene response factor in chlorophyll degradation during citrus fruit degreening. Plant J. 86, 403–412. 27037684 10.1111/tpj.13178

[pbi13272-bib-0061] Zhao, M.L. , Wang, J.N. , Shan, W. , Fan, J.G. , Kuang, J.F. , Wu, K. , Li, X.P. *et al.* (2012) Induction of jasmonate signaling regulators MaMYC2s and their physical interactions with MaICE1 in methyl jasmonate‐induced chilling tolerance in banana fruit. Plant Cell Environ. 36, 30–51. 22651394 10.1111/j.1365-3040.2012.02551.x

[pbi13272-bib-0062] Zhao, C. , Zhang, Z. , Xie, S. , Si, T. , Li, Y. and Zhu, J.K. (2016) Mutational evidence for the critical role of CBF transcription factors in cold acclimation in Arabidopsis. Plant Physiol. 171, 2744–2759. 27252305 10.1104/pp.16.00533 PMC4972280

[pbi13272-bib-0063] Zhou, M.Q. , Chen, H. , Wei, D.H. , Ma, H. and Lin, J. (2017) *Arabidopsis* CBF3 and DELLAs positively regulate each other in response to low temperature. Sci. Rep. 7, 39819. 28051152 10.1038/srep39819 PMC5209670

[pbi13272-bib-0064] Zhu, J.Y. , Oh, E. , Wang, T. and Wang, Z.Y. (2016) TOC1–PIF4 interaction mediates the circadian gating of thermoresponsive growth in *Arabidopsis* . Nat. Commun. 7, 13692. 27966533 10.1038/ncomms13692 PMC5171658

