# Supplementary material for: Crosstalk of PIF4 and DELLA modulates CBF transcript and hormone homeostasis in cold response in tomato
Source: Plant Biotechnol J. 2019 Oct 27;18(4):1041–55. doi: 10.1111/pbi.13272 (PMC7061876; doi:10.1111/pbi.13272)
Supplement: Supplementary file 1 — Figure S1 Phylogenetic analysis of tomato PIF family genes (SlPIFs). Figure S2 Low R/FR enhances the cold tolerance in tomato plants. Figure S3 Tomato pif4 mutant and SlPIF4‐overexpressing plants. Figure S4 SlPIF4 has no effect on the hypocotyl length in tomato plants. Figure S5 Phenotypes (a) and expression of COR413‐like gene (b) in tomato WT, pif4 mutants and SlPIF4‐OE plants after exposure to 25 °C or 4 °C for 7 days or 6 h, respectively, under high R/FR or low R/FR light conditions. Figure S6 ChIP‐qPCR assay shows the relative amount of SlCBF2 and SlCBF3 fragments in 35S: SlPIF4‐HA and wild‐type tomato plants. Figure S7 SlPIF4 positively regulates expression of ABA and JA signalling genes in response to cold stress. Figure S8 Phylogenetic analysis of tomato GAI family genes (SlGAIs) and schematic diagram showing vectors construction in dual‐luciferase assays. Figure S9 Expression of SlGAI4 gene in wild‐type (WT/pTRV), SlGAI4‐silenced plants (pTRV‐GAI4) and SlGAI4‐overexpressing plants (OE#54, OE#56). Figure S10 Phenotypes (a) and expression of COR413‐like gene in tomato SlGAI4‐silenced plants (pTRV‐GAI4; b) and SlGAI4‐overexpressing plants (OE#54, OE#56; c) after exposure to 25 °C or 4 °C for 7 days or 6 h, respectively, under high R/FR or low R/FR light conditions. Figure S11 SlGAI4 positively regulates expression of ABA and JA signalling genes in response to cold stress. Figure S12 The effects of GA3 and PAC on cold tolerance in tomato WT, pif4 mutant and SlPIF4‐OE plants. Figure S13 The effects of GA3 and PAC on cold tolerance in tomato WT and not plants. Figure S14 Expression of SlPIF4 in tomato SlGAI4‐silenced plants (a) and SlGAI4‐overexpressing plants (b) after exposure to 25 °C or 4 °C under H‐R/FR or L‐R/FR conditions for 6 h. Table S1 PCR primer sequences used for vector construction Table S2 List of primer sequences used for qRT‐PCR analysis Table S3 Probes used in the electrophoretic mobility shift assays (EMSA) Table S4 Primers used for ChIP‐qPCR a [file PBI-18-1041-s001.docx]

**Supporting Information**

**Article title:** Crosstalk of PIF4 and DELLA modulates CBF transcript and hormone homeostasis in cold response in tomato

**Authors:** Feng Wang, Xiaoxiao Chen, Sangjie Dong, Xiaochun Jiang, Lingyu Wang, Jingquan Yu and Yanhong Zhou

The following Supporting Information is available for this article:

**Figure S1** Phylogenetic analysis of tomato *PIF* family genes (*SlPIFs*).

**Figure S2** Low R/FR enhances the cold tolerance in tomato.

**Figure S3** Tomato *pif4* mutant and *SlPIF4*-overexpressing plants.

**Figure S4** SlPIF4 has no effect on the hypocotyl length in tomato.

**Figure S5** Phenotypes (a) and expression of *COR413-like* gene (b) in tomato WT, *pif4* mutants and *SlPIF4*-OE plants after exposure to 25 °C or 4 °C for 7 d or 6 h, respectively, under high R/FR or low R/FR light conditions.

**Figure S6** ChIP-qPCR assay shows the relative amount of *SlCBF2* and *SlCBF3* fragments in *35S: SlPIF4-*HA and wild-type tomato plants.

**Figure S7** SlPIF4 positively regulates expression of ABA and JA signaling genes in response to cold stress.

**Figure S8** Phylogenetic analysis of tomato *GAI* family genes (*SlGAIs*) and schematic diagram showing vectors construction in dual-luciferase assays.

**Figure S9** Expression of *SlGAI4* gene in wild-type (WT/pTRV), *SlGAI4*-silenced plants (pTRV-*GAI4*) and *SlGAI4*-overexpressing plants (OE#54, OE#56).

**Figure S10** Phenotypes (a) and expression of *COR413-like* gene in tomato *SlGAI4*-silenced plants (pTRV-*GAI4*; b) and *SlGAI4*-overexpressing plants (OE#54, OE#56; c) after exposure to 25 °C or 4 °C for 7 d or 6 h, respectively, under high R/FR or low R/FR light conditions.

**Figure S11** *SlGAI4* positively regulates expression of ABA and JA signaling genes in response to cold stress.

**Figure S12** The effects of GA_3_ and PAC on cold tolerance in tomato WT, *pif4* mutant and *SlPIF4*-OE plants.

**Figure S13** The effects of GA_3_ and PAC on cold tolerance in tomato WT and *not* plants.

**Figure S14** Expression of *SlPIF4* in tomato *SlGAI4*-silenced plants (a) and *SlGAI4*-overexpressing plants (b) after exposure to 25 °C or 4 °C under H-R/FR or L-R/FR conditions for 6 h.

**Table S1** PCR primer sequences used for vector construction.

**Table S2** List of primer sequences used for qRT-PCR analysis.

**Table S3** Probes used in the electrophoretic mobility shift assays (EMSA).

**Table S4** Primers used for ChIP-qPCR assays.


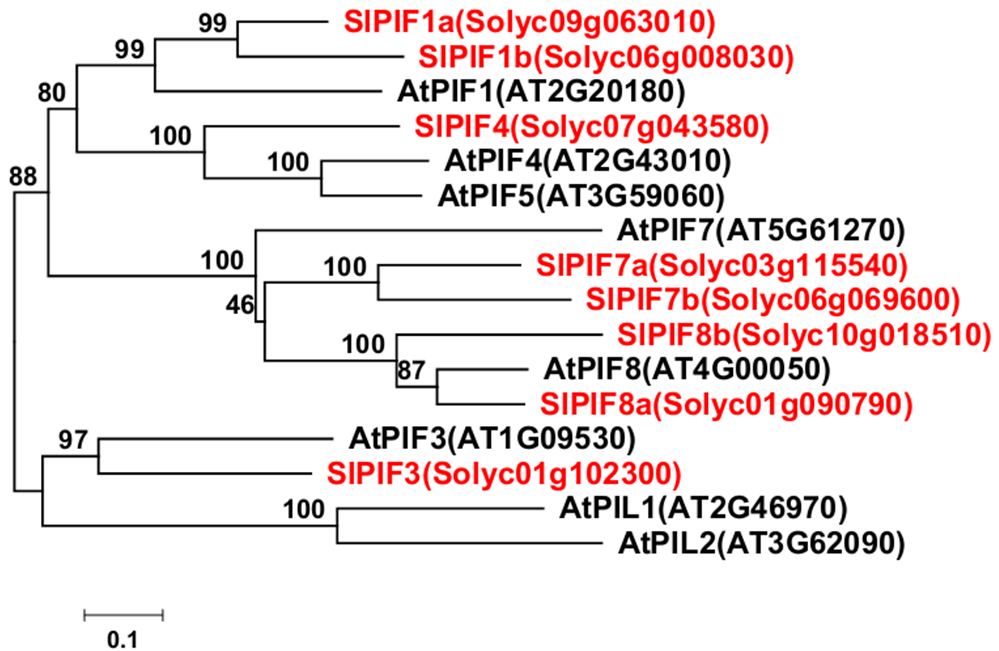


**Figure S1** Phylogenetic analysis of tomato *PIF* family genes (*SlPIFs*). Sequence alignments with different tomato (Solanum lycopersicum) reference sequences were from the Sol genomics network (available at: http://solgenomics.net/). The percentage at branch represents the posterior probabilities of amino acid sequences. Sequence alignment and tree construction were performed with the MEGA program. A consensus neighbor-joining tree was obtained from 1000 bootstrap replicates of aligned sequences.


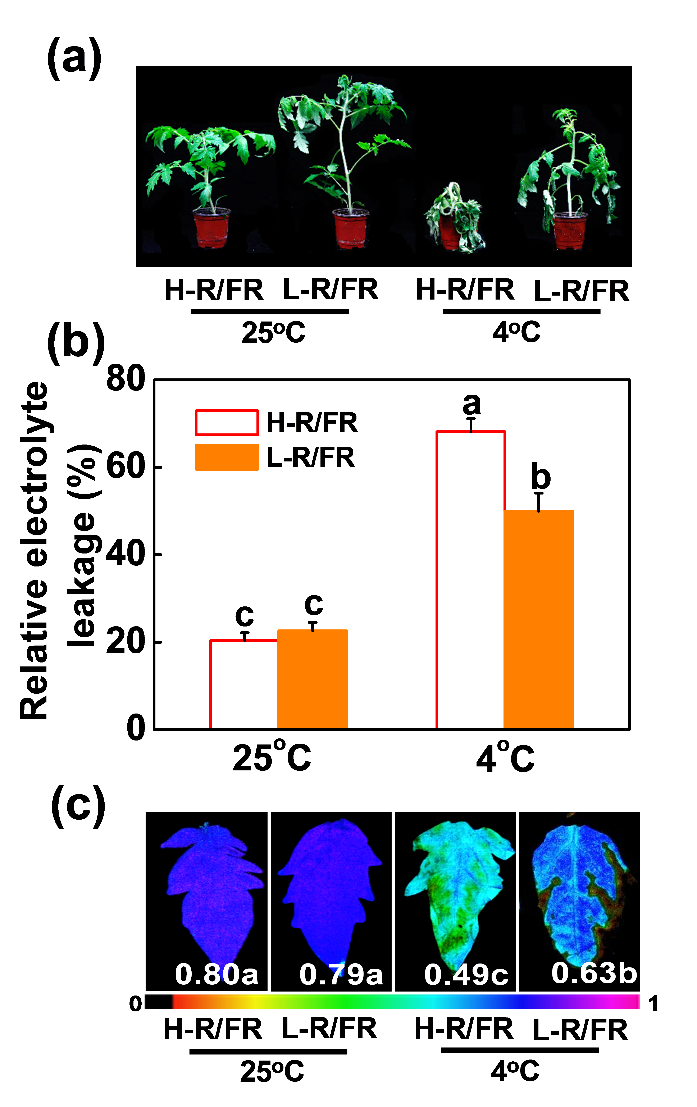


**Figure S2** Low R/FR enhances the cold tolerance in tomato plants. **(**a-c) Phenotypes (a), relative electrolyte leakage (b) and Fv/Fm (c) in tomato plants after exposure to 25 °C or 4 °C under high R/FR (H-R/FR, 4.5) light or low R/FR (L-R/FR, 0.5) light for 7 d. The false-color code depicted at the bottom of the image ranges from 0 (black) to 1.0 (purple), representing the level of damage in the leaves. For light-quality treatments, plants were maintained at white light conditions (120 µmol m^-2^ s^-1^) and supplemented with different intensities of FR. Data are presented as the means of three biological replicates (±SD). Different letters indicate significant differences (*P* < 0.05) according to Tukey’s test.


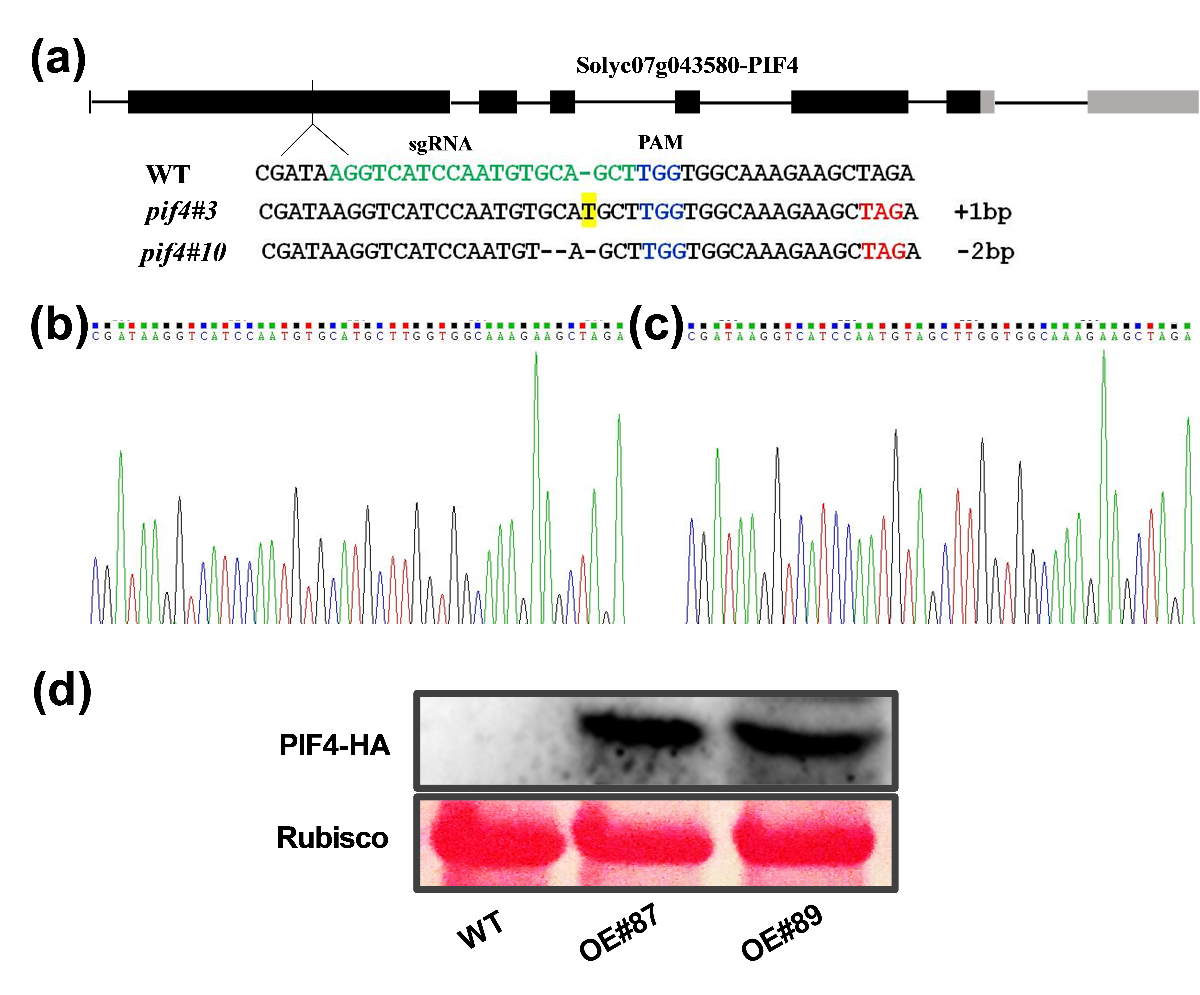


**Figure S3** Tomato *pif4* mutant and *SlPIF4*-overexpressing plants. (a) The diagram displays the structure of the *SlPIF4* gene. The *SlPIF4* gene has six exons (black closed boxes) and seven introns (black horizontal lines). PAM is indicated by blue, single guide RNA (sgRNA) is indicated by green, one insert mutation of a single nucleotide is indicated by yellow, and stop codon is in red. The dashed lines represent nucleotide deletions. (b) and (c) Sequencing results in *pif4*#3 (b) and *pif4*#10 (c) mutants. (d) Accumulation of SlPIF4-HA protein in tomato wild-type (WT) and *SlPIF4*-overexpressing (*SlPIF4-*OE#87, OE#89) plants. Samples are from the 4^th^ leaf of *SlPIF4*-overexpressing transgenic tomato plants at 4-leaf stage.


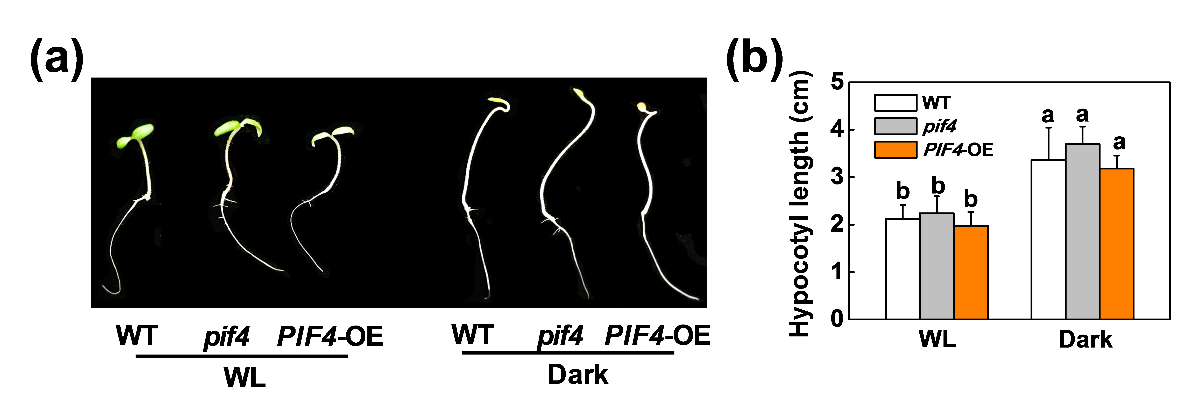


**Figure S4** SlPIF4 has no effect on the hypocotyl length in tomato plants. **(**a) and (b) Phenotypes (a) and hypocotyl length (b) in tomato plants after the germination seeding exposure to white light (WL, 12 h light/12 h dark) or dark (24 h dark) conditions for 7 days. The WL intensity was 120 µmol m^-2^ s^-1^. Data are presented as the means of three biological replicates (±SD). Different letters indicate significant differences (*P* < 0.05) according to Tukey’s test.


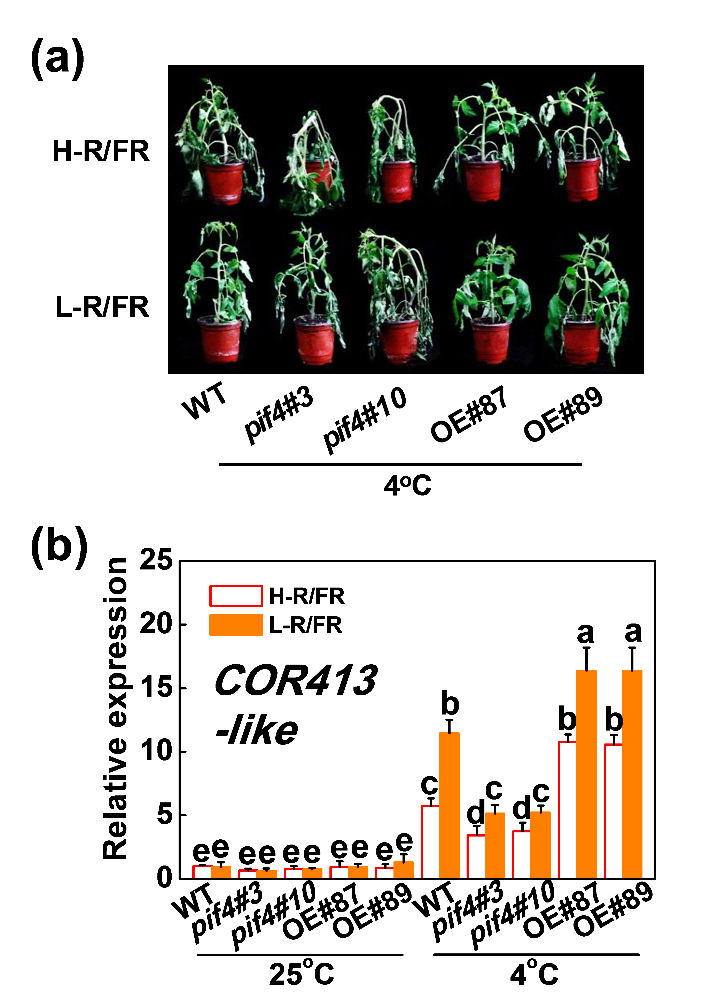


**Figure S5** Phenotypes (a) and expression of *COR413-like* gene (b) in tomato WT, *pif4* mutant and *SlPIF4*-OE plants after exposure to 25 °C or 4 °C for 7 d or 6 h, respectively, under high R/FR or low R/FR light conditions. For light-quality treatments (H-R/FR, 2.5; L-R/FR, 0.5), plants were maintained at R conditions (120 µmol m^-2^ s^-1^) and supplemented with different intensities of FR. Data are presented as the means of three biological replicates (±SD). Different letters indicate significant differences (*P* < 0.05) according to Tukey’s test.


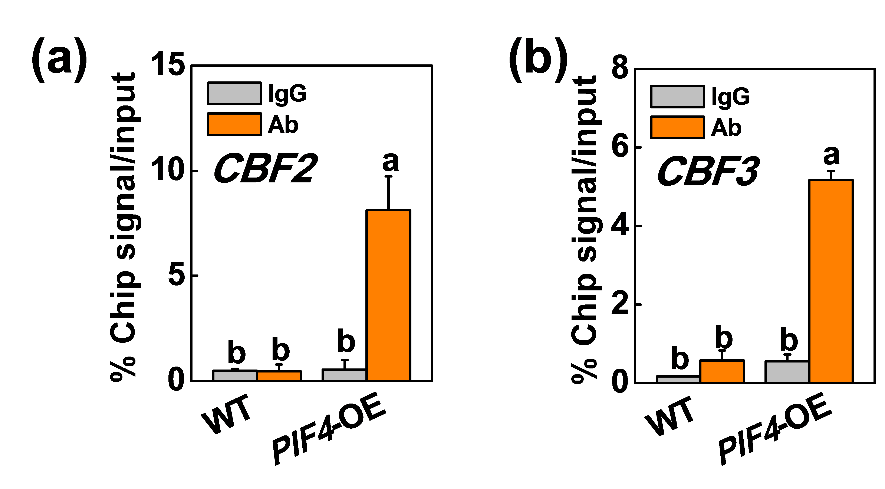


**Figure S6** ChIP-qPCR assay shows the relative amount of *SlCBF2* (a) and *SlCBF3* (b) fragments in *35S: SlPIF4-*HA and wild-type tomato plants. Plants were grown at 4 °C under L-R/FR (0.5) light condition for 6 h, and samples were precipitated with an anti-HA antibody. A control reaction was processed simultaneously using mouse IgG. The ChIP results are presented as percentages of the input DNA. For light-quality treatments, plants were maintained at R conditions (120 µmol m^-2^ s^-1^) and supplemented with different intensities of FR. Data are presented as the means of three biological replicates (±SD). Different letters indicate significant differences (*P* < 0.05) according to Tukey’s test.

**
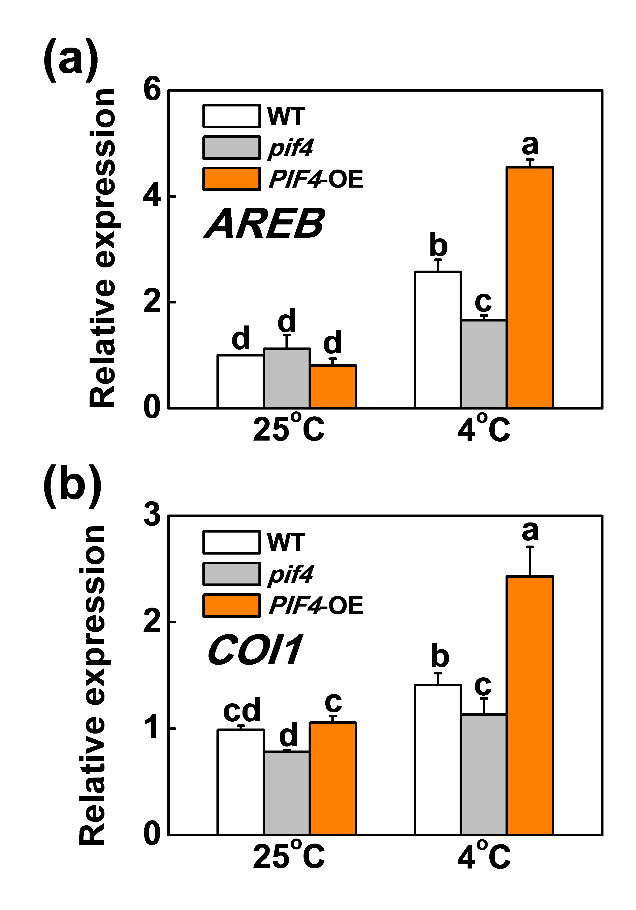
**

**Figure S7** SlPIF4 positively regulates expression of ABA and JA signaling genes in response to cold stress. (a) and (b) Expression of *AREB* (a) and *COI1* (b) in tomato WT, *pif4* mutant and *SlPIF4*-OE plants after exposure to 25 °C or 4 °C under low R/FR (L-R/FR, 0.5) light conditions for 6 h. For light-quality treatments, plants were maintained at R conditions (120 µmol m^-2^ s^-1^) and supplemented with different intensities of FR. Data are presented as the means of three biological replicates (±SD). Different letters indicate significant differences (*P* < 0.05) according to Tukey’s test.

**
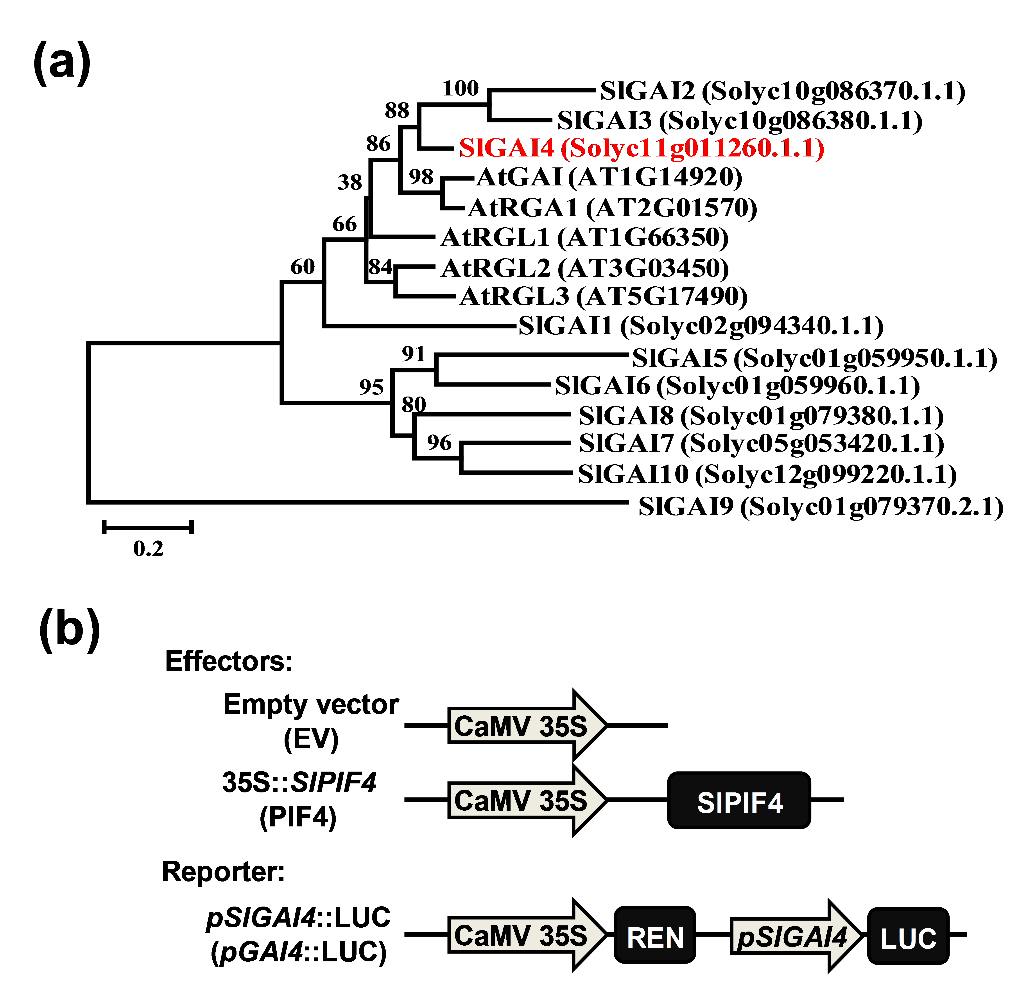
**

**Figure** **S8** Phylogenetic analysis of tomato *GAI* family genes (*SlGAIs*) and schematic diagram showing vectors construction in dual-luciferase assays. (a) Sequence alignments with different tomato (Solanum lycopersicum) reference sequences were from the Sol genomics network (available at: http://solgenomics.net/). The percentage at branch represents the posterior probabilities of amino acid sequences. Sequence alignment and tree construction were performed with the MEGA program. A consensus neighbor-joining tree was obtained from 1000 bootstrap replicates of aligned sequences. (b) Vectors construct in dual-luciferase assays. The *SlGAI4* promoter was fused to the luciferase (LUC) reporter (pGAI4::LUC), and promoter activity was determined by transient expression of it with empty vector (EV) or 35S:*SlPIF4* (PIF4) in tobacco.


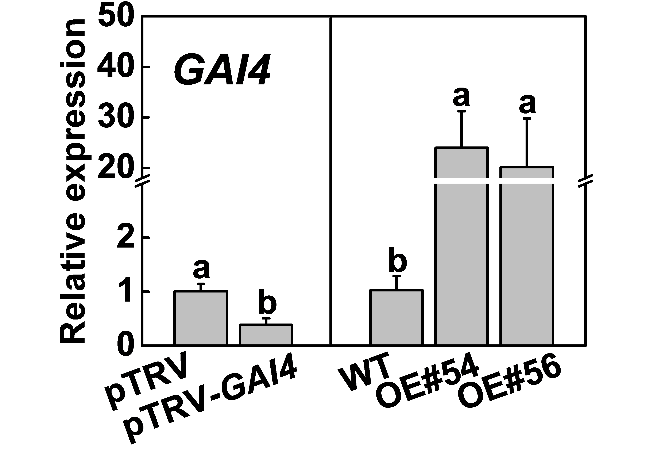


**Figure S9** Expression of *SlGAI4* gene in wild-type (WT/pTRV), *SlGAI4*-silenced plants (pTRV-*GAI4*) and *SlGAI4*-overexpressing plants (OE#54, OE#56). Samples are from the 4^th^ leaf of tomato plants at 4-leaf stage. Data are the means (±SD) of three biological replicates. Different letters indicate significant differences (*P*< 0.05) according to the Tukey’s test.


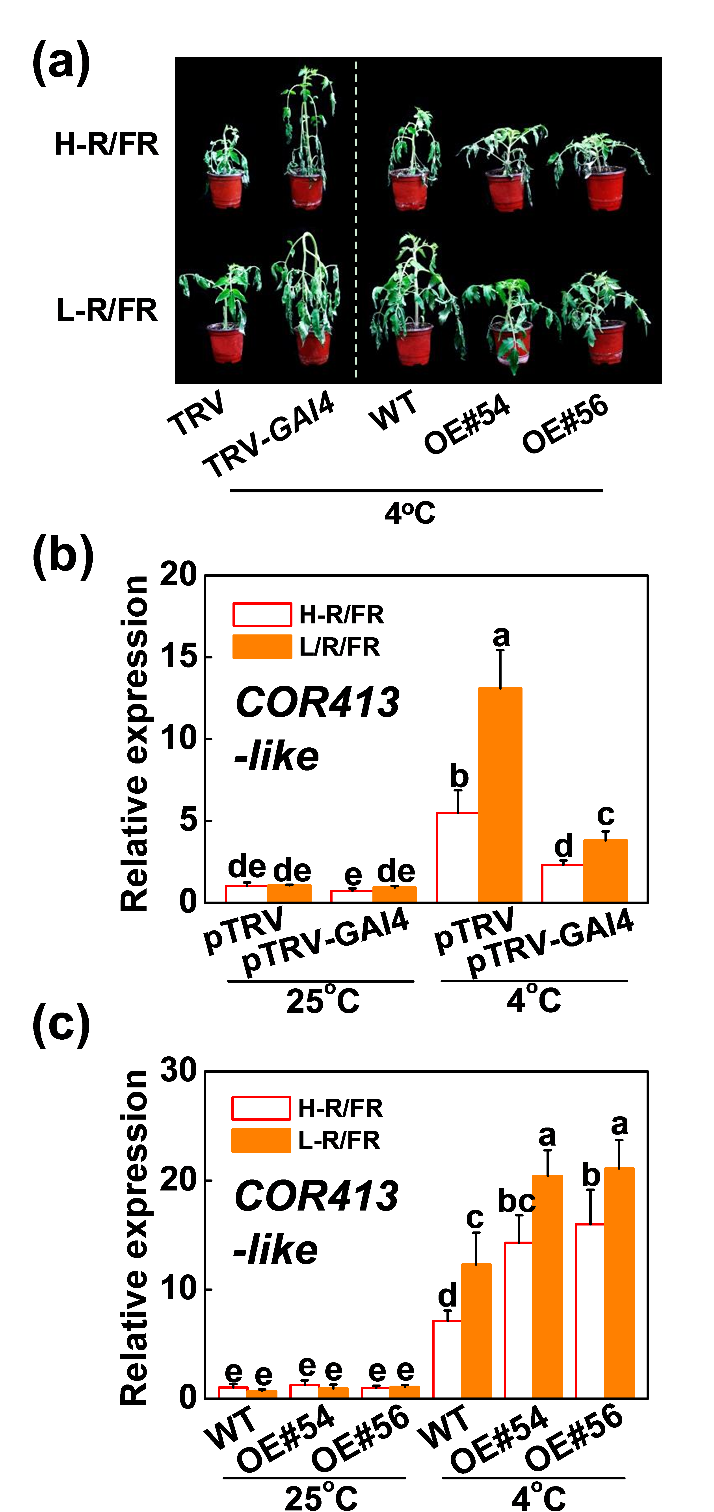


**Figure S10** Phenotypes (a) and expression of *COR413-like* gene in tomato *SlGAI4*-silenced plants (pTRV-*GAI4*; b) and *SlGAI4*-overexpressing plants (OE#54, OE#56; c) after exposure to 25 °C or 4 °C for 7 d or 6 h, respectively, under high R/FR or low R/FR light conditions. For light-quality treatments (H-R/FR, 2.5; L-R/FR, 0.5), plants were maintained at R conditions (120 µmol m^-2^ s^-1^) and supplemented with different intensities of FR. Data are presented as the means of three biological replicates (±SD). Different letters indicate significant differences (*P* < 0.05) according to Tukey’s test.


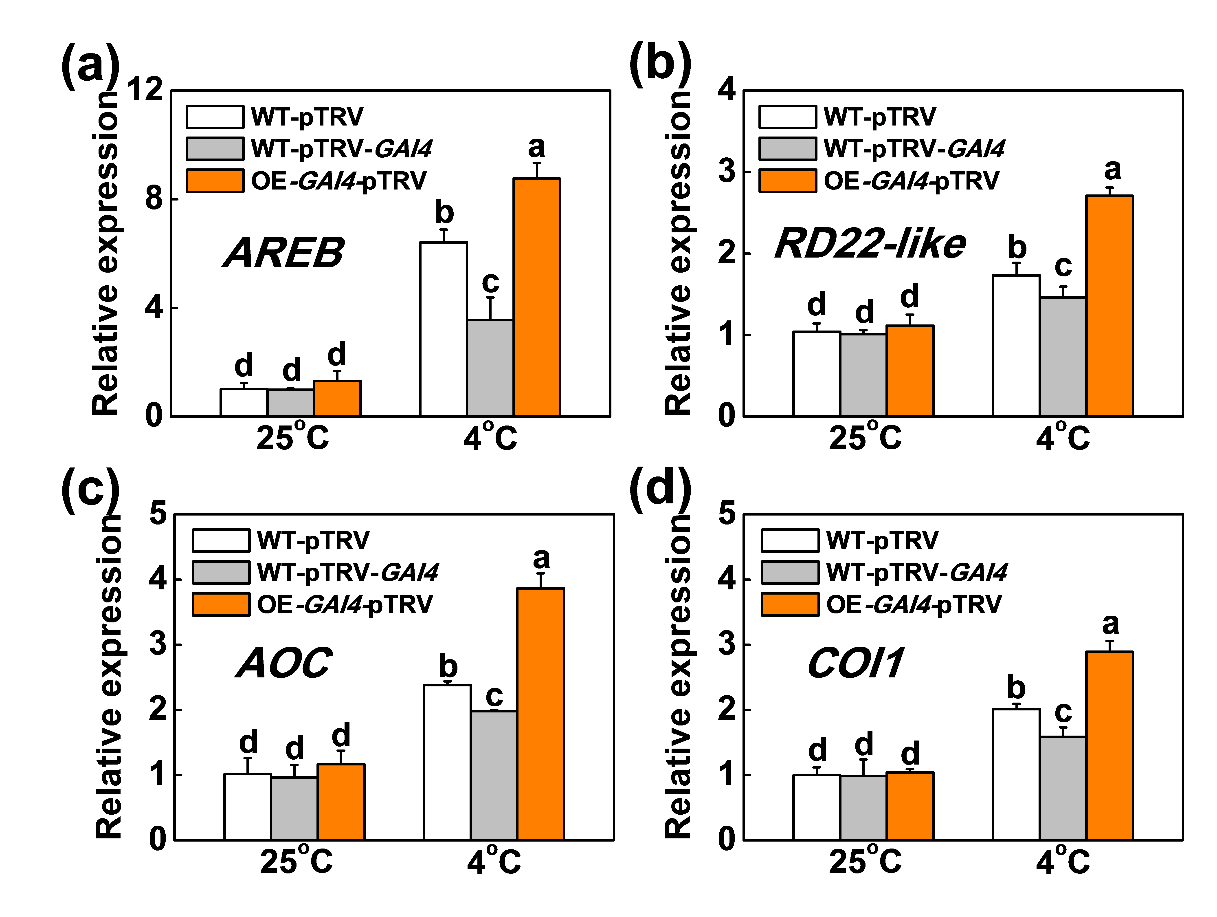


**Figure S11** *SlGAI4* positively regulates expression of ABA and JA signaling genes in response to cold stress. (a) to (d) Expression of *AREB* (a), *RD22-like* (b), *AOC* (c) and *COI1* (d) in tomato wild-type (WT-pTRV), *SlGAI4*-silenced plants (WT-pTRV-*GAI4*) and *SlGAI4* overexpressing plants (OE-*GAI4*-pTRV) after exposure to 25 °C or 4 °C under low R/FR (L-R/FR, 0.5) light conditions for 6 h. For light-quality treatments, plants were maintained at R conditions (120 µmol m^-2^ s^-1^) and supplemented with different intensities of FR. Data are presented as the means of three biological replicates (±SD). Different letters indicate significant differences (*P* < 0.05) according to Tukey’s test.


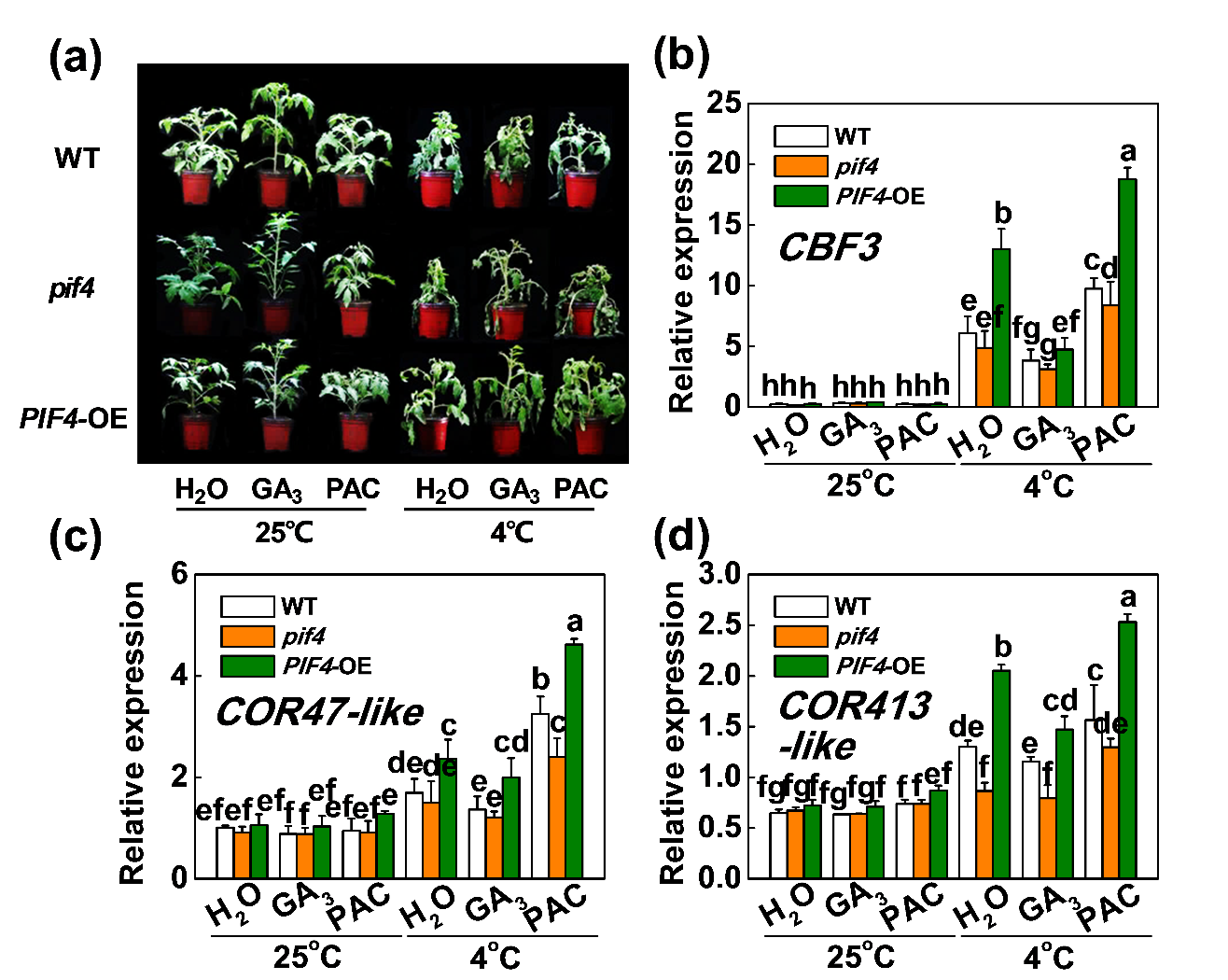


**Figure S12** The effects of GA_3_ and PAC on cold tolerance in tomato WT, *pif4* mutant and *SlPIF4*-OE plants. (a) Phenotypes in tomato wild-type (WT), *pif4* mutant (*pif4*) and *SlPIF4-*overexpressing plants (*SlPIF4*-OE) after exposure to 25 °C or 4 °C under low R/FR (L-R/FR, 0.5) light conditions for 7 d, which pretreated with water (H_2_O), GA_3_ (50 μM) or paclobutrazol (PAC, GA biosynthesis inhibitor, 25 μM) for 12 h prior to exposure to cold conditions at 4 °C. (b) to (d) Expression of *SlCBF3* (b)*,* *COR47-like* (c) and *COR413-like* (d) in tomato WT, *pif4* and *SlPIF4*-OE plants after exposure to 25 °C or 4 °C under L-R/FR light conditions for 6 h, which pretreated with H_2_O, GA_3_ or PAC for 12 h prior to exposure to cold conditions at 4 °C. For light-quality treatments, plants were maintained at R conditions (120 µmol m^-2^ s^-1^) and supplemented with different intensities of FR. Data are presented as the means of three biological replicates (±SD). Different letters indicate significant differences (*P* < 0.05) according to Tukey’s test.


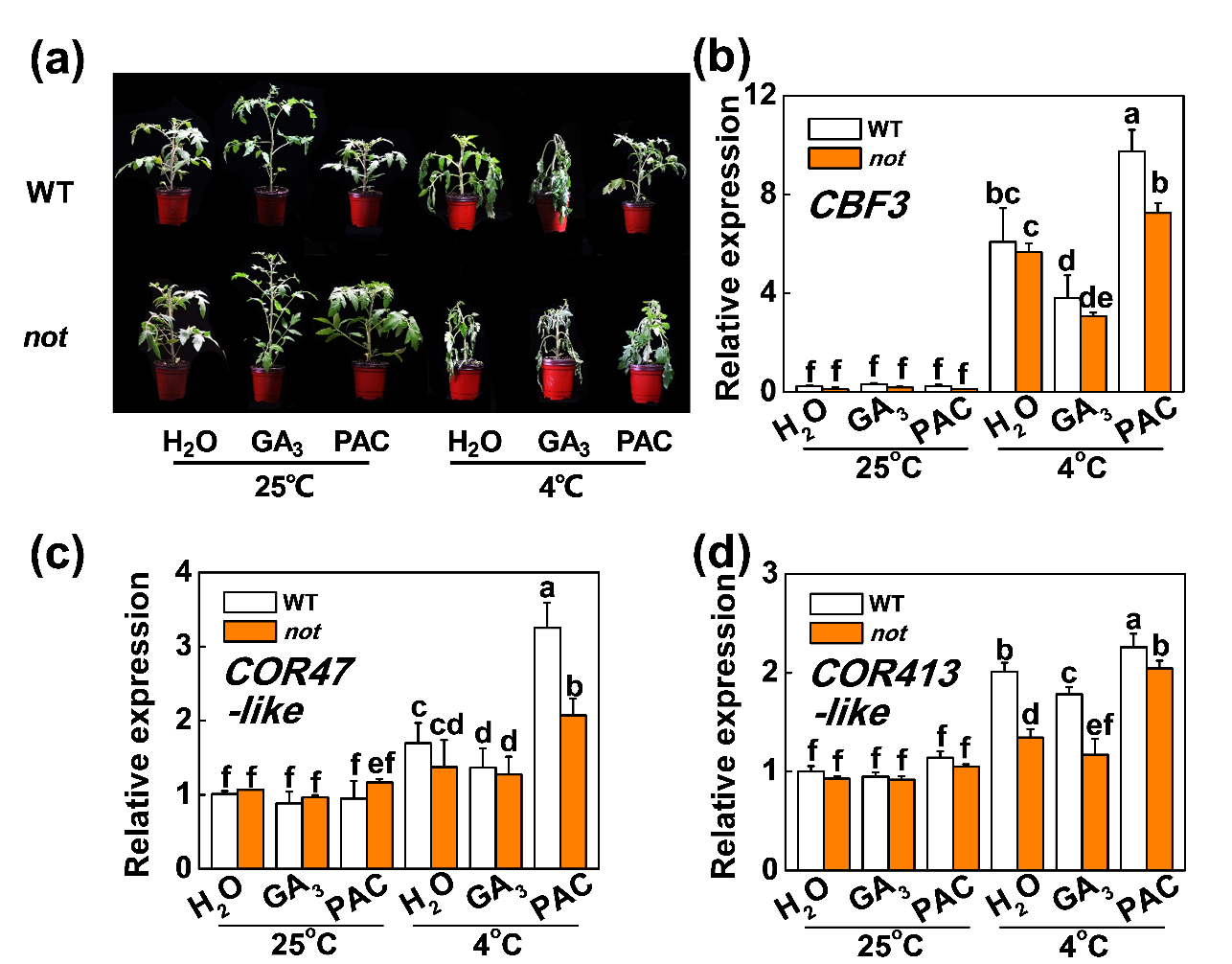


**Figure S13** The effects of GA_3_ and PAC on cold tolerance in tomato WT and *not* plants. (a) Phenotypes in tomato wild-type (WT) and ABA-deficient mutant (*not*) after exposure to 25 °C or 4 °C under low R/FR (L-R/FR, 0.5) light conditions for 7 d, which pretreated with water (H_2_O), GA_3_ (50 μM) or paclobutrazol (PAC, GA biosynthesis inhibitor, 25 μM) for 12 h prior to exposure to cold conditions at 4 °C. (b) to (d) Expression of *SlCBF3* (b)*,* *COR47-like* (c) and *COR413-like* (d) in tomato WT and *not* plants after exposure to 25 °C or 4 °C under L-R/FR light conditions for 6 h, which pretreated with H_2_O, GA_3_ or PAC for 12 h prior to exposure to cold conditions at 4 °C. For light-quality treatments, plants were maintained at R conditions (120 µmol m^-2^ s^-1^) and supplemented with different intensities of FR. Data are presented as the means of three biological replicates (±SD). Different letters indicate significant differences (*P* < 0.05) according to Tukey’s test.


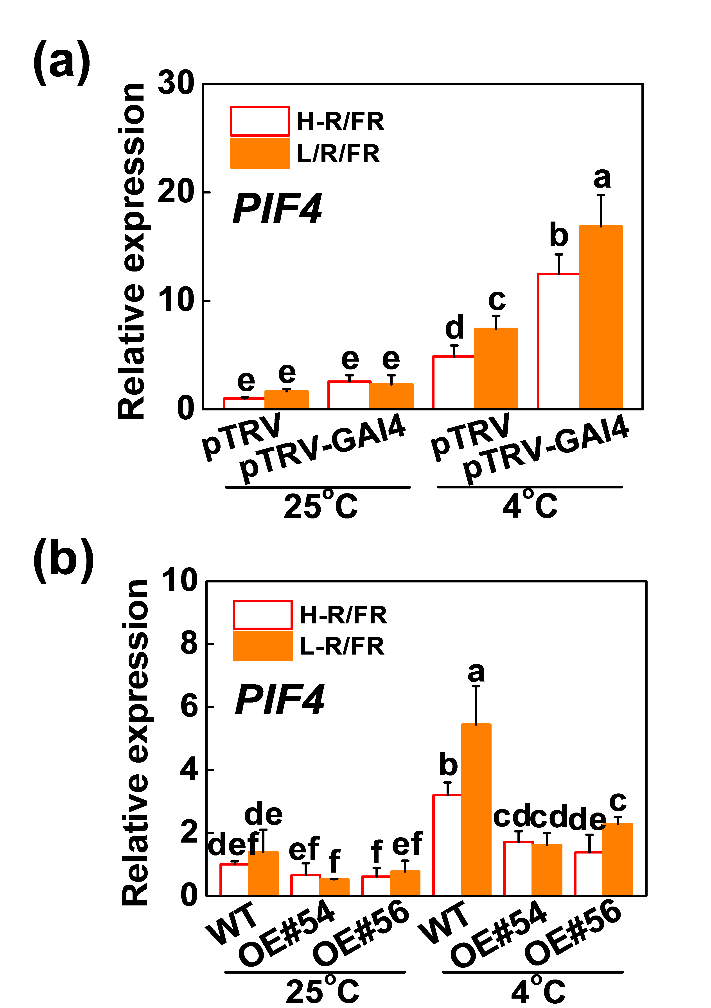


**Figure** **S14** Expression of *SlPIF4* in tomato *SlGAI4*-silenced plants (a) and *SlGAI4*-overexpressing plants (b) after exposure to 25 °C or 4 °C under H-R/FR or L-R/FR conditions for 6 h. For light-quality treatments (H-R/FR, 2.5; L-R/FR, 0.5), plants were maintained at R conditions (120 µmol m^-2^ s^-1^) and supplemented with different intensities of FR. Data are presented as the means of three biological replicates (±SD). Different letters indicate significant differences (*P* < 0.05) according to Tukey’s test.

**Table S1 PCR primer sequences used for vector construction.**

| **Vector** | **primer** | |
| --- | --- | --- |
| **OE-*PIF4*** | **Forward** | 5′-TTGgcgcgccATGAATCCATATCTTCCT-3′ |
|  | **Reverse** | 5′-GCgtcgacAGTGGCAGGTGCATTACTAT-3′ |
| ***pif4*** | **Forward** | 5′-gattgAGGTCATCCAATGTGCAGCT-3′ |
|  | **Reverse** | 5′-aaacAGCTGCACATTGGATGACCTc-3′ |
| **pET-32a-*PIF4*** | **Forward** | 5′-CGCggatccATGAATCCATATCTTCCTGA-3′ |
|  | **Reverse** | 5′-CGgagctcTCAAGTGGCAGGTGCATTAC-3′ |
| **SK-*PIF4*** | **Forward** | 5′-GCtctagaATGAATCCATATCTTCCTGA-3′ |
|  | **Reverse** | 5′-CGCggatccTCAAGTGGCAGGTGCATTAC-3′ |
| ***GAI4*-LUC** | **Forward** | 5′-ACGCgtcgacCTATAATAAGGTCCTATTATC-3′ |
|  | **Reverse** | 5′-CGggatccCTTTTAAGAGATGGGGTTCAA-3′ |
| **OE-*GAI4*** | **Forward** | 5′-TTGgcgcgccATGAAGAGAGATCGAGATCG-3′ |
|  | **Reverse** | 5′-ACGCgtcgacCAACTCGACTTCTCCGGCGC-3′ |
| **pTRV2-*GAI1*** | **Forward** | 5′-CCGgaattcGAGAGCTTTAACCTGCCAGC-3′ |
|  | **Reverse** | 5′-GCtctagaAAGCTAGCGGACGTATTGGA-3′ |
| **pTRV2-*GAI2*** | **Forward** | 5′-CCGgaattcCATGGTGGTTTATTGCCGGA-3′ |
|  | **Reverse** | 5′-GCtctagaATCTGCAAAATGTGTCGCCA-3′ |
| **pTRV2-*GAI3*** | **Forward** | 5′-CCGgaattcTGTTGATCTCAGGGCACTTTG-3′ |
|  | **Reverse** | 5′-GCtctagaTGCTCCTCCCATTTGTGAAAC-3′ |
| **pTRV2-*GAI4*** | **Forward** | 5′-CCGgaattcCAGATGCTGGAATGGATGAGC-3′ |
|  | **Reverse** | 5′-GCtctagaACTTTGTACCCAACCAGCCA-3′ |
| **pTRV2-*GAI5*** | **Forward** | 5′-CCGgaattcTGCAGTGGTGTCAGAAATGA-3′ |
|  | **Reverse** | 5′-GCtctagaTGGCTCGTTAATGTTTGCCT-3′ |
| **pTRV2-*GAI6*** | **Forward** | 5′-CCGgaattcCAAGAGGACAACTGTGAGCA-3′ |
|  | **Reverse** | 5′-GCtctagaAAAGGCCCAAATCCGCAATT-3′ |
| **pTRV2-*GAI7*** | **Forward** | 5′-CCGgaattcTCGTTTACCCTTCGTGGTGA-3′ |
|  | **Reverse** | 5′-GCtctagaTCAAACTGTTCCTGGCCTACT-3′ |
| **pTRV2-*GAI8*** | **Forward** | 5′-CCGgaattcACAGGAAGCAATGTGGAGGA-3′ |
|  | **Reverse** | 5′-CGCggatccATCTTTCTCCGGCTAGCCTC-3′ |
| **pTRV2-*GAI9*** | **Forward** | 5′-CGCggatccTTTCTAGGCTATCTGTTGCACA-3′ |
|  | **Reverse** | 5′-GCtctagaAGGTATGAGAAAGTGCAGCAAC-3′ |
| **pTRV2-*GAI10*** | **Forward** | 5′-CCGgaattcTTGGGATTTCGACGTTGTAGA-3′ |
|  | **Reverse** | 5′-GCtctagaTGCATAAACTTTTGTCCGCCA-3′ |
| **pTRV2-*PIF1a*** | **Forward** | 5′-CCGgaattcGCCCAACTTCTTGCTTTGCT-3′ |
|  | **Reverse** | 5′-GCtctagaGCTCACACACAGAAAAGGCT-3′ |
| **pTRV2-*PIF1b*** | **Forward** | 5′-CCGgaattcACTGTGTTGTTCCTGATTTCAAA-3′ |
|  | **Reverse** | 5′-GCtctagaAGATCTTTGATTTTGGCTTTGCA-3′ |
| **pTRV2-*PIF3*** | **Forward** | 5′-CCGgaattcGGGGTTTGACTGTGTTACTGG-3′ |
|  | **Reverse** | 5′-GCtctagaATGAGATCTTCTGCTGGCCA-3′ |
| **pTRV2-*PIF4*** | **Forward** | 5′-CCGgaattcGCTAGTGGAGTTGTTGTGGC-3′ |
|  | **Reverse** | 5′-GCtctagaCCACGCGACAGTTTCATCAT-3′ |
| **pTRV2-*PIF7a*** | **Forward** | 5′-CCGgaattcTGTGAAGAAGTTGCAGAGCTG-3′ |
|  | **Reverse** | 5′-GCtctagaTTCGCGTGATATGTGGCTTG-3′ |
| **pTRV2-*PIF7b*** | **Forward** | 5′-CCGgaattcCGGAAGCAACCTCTCGAATG-3′ |
|  | **Reverse** | 5′-GCtctagaCCCTGTTGATGTTTGCCTGA-3′ |
| **pTRV2-*PIF8a*** | **Forward** | 5′-CCGgaattcGGACCTCGATGACTCTACCG-3′ |
|  | **Reverse** | 5′-GCtctagaCGGGTGGCTTGGTTCACTAT-3′ |
| **pTRV2-*PIF8b*** | **Forward** | 5′-CCGgaattcATGCATGGGTTAGGTCCTCC-3′ |
|  | **Reverse** | 5′-GCtctagaTCATCATCACCACCCTCGTC-3′ |

The restriction enzyme cutting sites were indicated in red.

**Table S2 List of primer sequences used for qRT-PCR analysis.**

| **Gene** | **Accession number** | **Forward primer (5’-3’)** | **Reverse primer (5’-3’)** | |  |
| --- | --- | --- | --- | --- | --- |
| ***PIF1a*** | Solyc09g063010 | TCGTGGCTTCATTACCCACT | | GACGTACGGTTTTCTCGTGG | |
| ***PIF1b*** | Solyc06g008030 | CATCCAGAATCCCAGTTGCG | | CCACTTCCTGATTCTGGGGT | |
| ***PIF3*** | Solyc01g102300 | AGATGCCACATTTCTCCCCA | | TGCACAGAGGGCATTGGATA | |
| ***PIF4*** | Solyc07g043580 | TCCAATGCCACCTCCAAGAT | | AAGCTGCACATTGGATGACC | |
| ***PIF7a*** | Solyc03g115540 | ATTCTGTTAGCCCTGCCAGT | | TGCTGCCTGTGATCTGTGTA | |
| ***PIF7b*** | Solyc06g069600 | TCCTCCCTTCATGTCACCAC | | TGAGTTTGGGGCTGATGGAT | |
| ***PIF8a*** | Solyc01g090790 | TGCTGCTATGCCTGATCCTT | | CAGGTACAATGCTGCCATCC | |
| ***PIF8b*** | Solyc10g018510 | GCAGCCCCAACATAACAACA | | GGTACAATGCTGCCATCCTG | |
| ***GAI1*** | Solyc02g094340 | TCTTTGATTCCCTGGACGCT | | CCATCACCCTCCTCCACTTT | |
| ***GAI2*** | Solyc10g086370 | CGAATTGTGAGACGTGGGAC | | GCATTTGGGAAAGAGGCCAA | |
| ***GAI3*** | Solyc10g086380 | CGATAGCTGCCTCTTCCTCA | | CGAATTCCTGTGAGCCGAAG | |
| ***GAI4*** | Solyc11g011260 | TGGGTCTTCGTCTTCAGCTT | | CACAAGCCACCACGTTACAA | |
| ***GAI5*** | Solyc01g059950 | ACAACGTCAGGCTGGAAGTA | | CCAGAGTGCAAAAGCCATGA | |
| ***GAI6*** | Solyc01g059960 | CGCCATGAGAAGCTTCGATC | | CTAGACTGCAAAGCCCATGG | |
| ***GAI7*** | Solyc05g053420 | AAGTAGACGTATGGAGGGCG | | TGTGCAACAACTCCCACAAG | |
| ***GAI8*** | Solyc01g079380 | GCACTTGGAGAGAGGATCGA | | TGCTACGAATGAGGCATGGA | |
| ***GAI9*** | Solyc01g079370 | CTCACCAACACAGTCATGCC | | TGGTACTTGAGGCGGGAAAT | |
| ***GAI10*** | Solyc12g099220 | GAGAAAGTCGGGGAGAAGGT | | TTTCGTGCAATTCTTCCCCG | |
| ***PHYA*** | Solyc10g044670 | AGACAAAGTTACCACCGCCTAC | | CAGTCCCGATGCCAAGAA | |
| ***PHYB1*** | Solyc01g059870 | AAGAGGCGTGGAAGGT | | CTGCCCAACAAAGGAA | |
| ***PHYB2*** | Solyc05g053410 | AGAACCGCAGTTTACC | | AGCAGTCTCCACCAAT | |
| ***CBF1*** | Solyc03g026280 | GAGTCGGAAGAAGTTTCAGG | | TGTAGGCATCAGTTTCCAC | |
| ***CBF3*** | Solyc03g026270 | TGCCGGGTTTACTTACGAAT | | TCAGCTTCCACATGATCTCC | |
| ***COR47-like*** | Solyc04g082200 | TCTAGTAGCTCCAGTGATG | | TCTCCTCTGTTTCCTCGT | |
| ***COR413-like*** | Solyc02g071210 | AGCAGCTTATCTGGGTTT | | AATGATGGCTAGGGTGGC | |
| ***NCED6*** | Solyc05g053530 | GGTCTTGAAGTTGTGGGTC | | CGGCTGGTTTGAGTGA | |
| ***AREB*** | Solyc04g078840 | TCCAGCACTCAACTCT | | TGGCTCCTAAACCTAC | |
| ***RD22-like*** | Solyc02g062320 | TGGAGGAAGAACAAAGAC | | TTGGTGGCATACTGATAG | |
| ***LOXD*** | Solyc03g122340 | GACTGGTCCAAGTTCACGATCC | | ATGTGCTGCCAATATAAATGGTTCC | |
| ***AOS2*** | Solyc11g069800 | ACCGGGACCATTCATCACTT | | GGTTTGGTTCAGATGGGTCG | |
| ***AOC*** | Solyc02g085730 | GCACGAAGAAGAGAAGAAAGGAGAT | | CGGTGACGGCTAGGTAAGTITC | |
| ***COI1*** | Solyc05g052620 | ATGGGCGAGCCATCGCTAAG | | AGCCTTGGCTACCTTGCAGC | |
| ***GA3ox2*** | Solyc03g119910 | CGGGTTGTACCCGAGTGTTC | | CCCATATAGATAGGCCACTGAAAGA | |
| ***GA20ox1*** | Solyc03g006880 | CTCATTGTGATCCAACATCATTAACA | | CCATAAATGTGTCGCCTATATTAACC | |
| ***ACTIN2*** | Solyc11g005330 | TGTCCCTATTTACGAGGGTTATGC | | CAGTTAAATCACGACCAGCAAGAT | |

**Table S3 Probes used in the electrophoretic mobility shift assays (EMSA).**

| **Gene** | **Accession number** | **Forward primer (5’-3’)** | **Reverse primer (5’-3’)** |
| --- | --- | --- | --- |
| ***CBF1*** | Solyc03g026280 | CTCAATTTTACACGTGTGTCCGCCTA | TAGGCGGACACACGTGTAAAATTGAG |
| ***GAI4*** | Solyc11g011260 | ATCACGTGTCACGTGGTTAAA | TTTAACCACGTGACACGTGAT |

**Table S4 Primers used for ChIP-qPCR assays.**

| **Gene** | **Accession number** | **Forward primer (5’-3’)** | **Reverse primer (5’-3’)** |
| --- | --- | --- | --- |
| ***CBF1*** | Solyc03g026280 | AAATGATGCACGTGGTCCAC | CTCAACACACGCTTTTGACG |
| ***CBF2*** | Solyc03g124110 | TGCCACCAAATAATGACACGT | ACGGCTTGAAATTTGCAACG |
| ***CBF3*** | Solyc03g026270 | CCAAGTTAGACGCACGGAAG | ATAGAGTGGGGCAGCTGTTT |
| ***GAI4*** | Solyc11g011260 | ACTATCTTGTGGGCCCAACG | CACAAGTACCCCTTCAGCCT |
